# Supplementary figures and images for: Comparison of miRNA expression profiles in pituitary–adrenal axis between Beagle and Chinese Field dogs after chronic stress exposure
Source: PeerJ. 2016 Feb 18;4:e1682. doi: 10.7717/peerj.1682 (PMC4768678; doi:10.7717/peerj.1682)

**BAC1**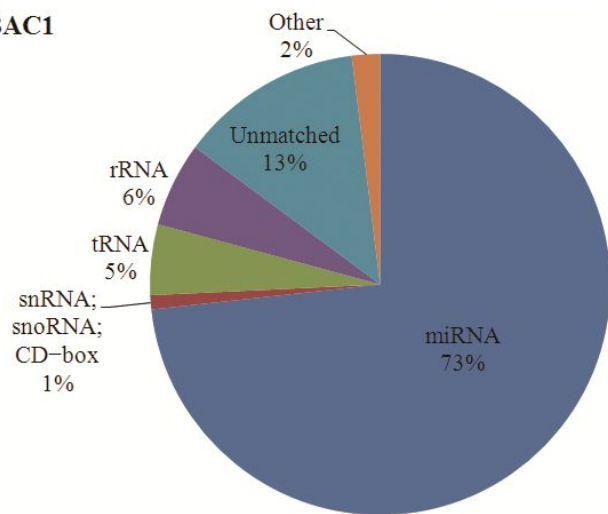**BAC2**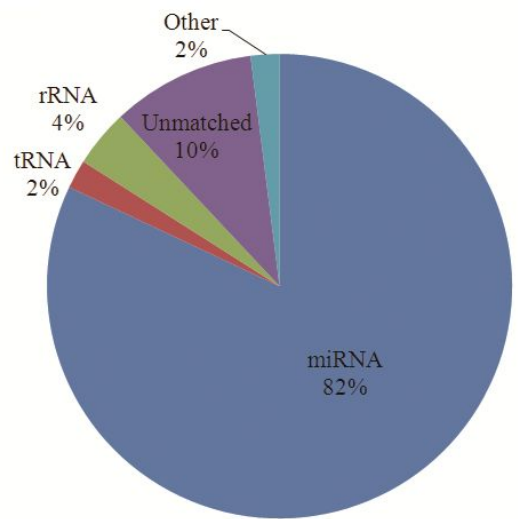**CFDAC1**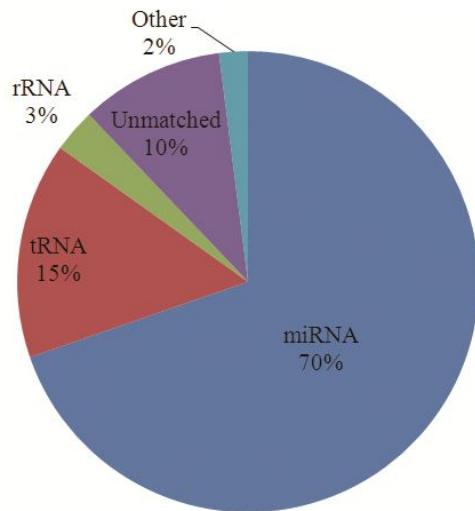**CFDAC2**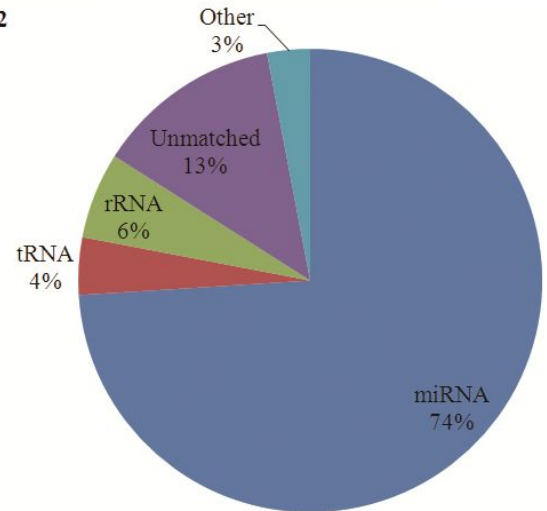**BP1**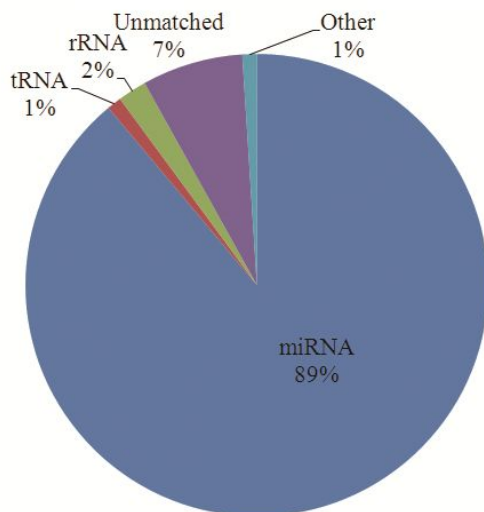**BP2**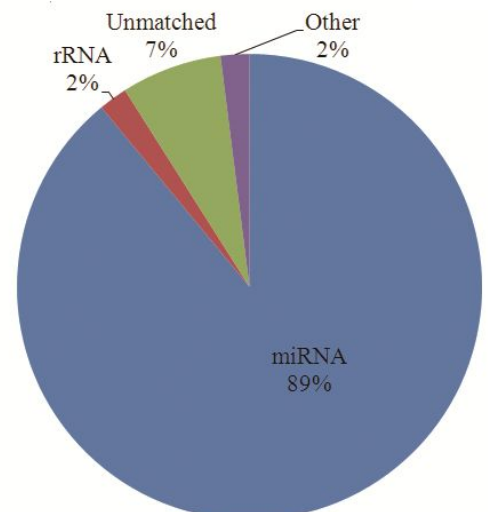**CFDP1**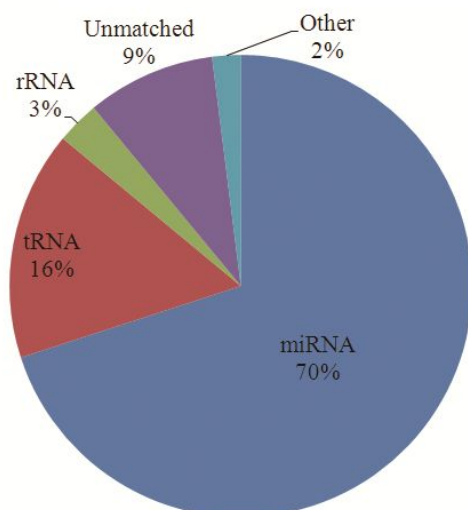**CFDP2**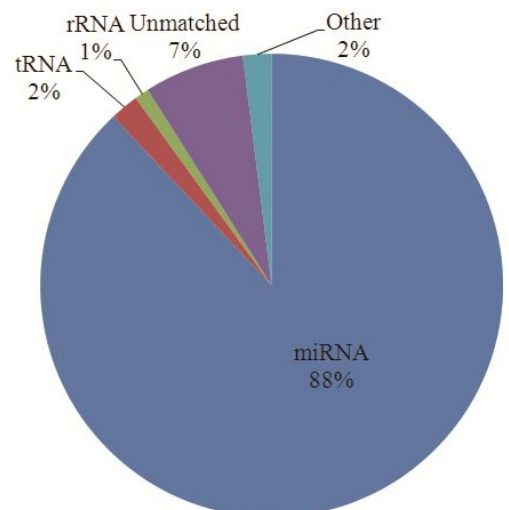

Supplement: Figure S1 [file peerj-04-1682-s001.pdf]
