# Supplementary material for: Comparison of miRNA expression profiles in pituitary–adrenal axis between Beagle and Chinese Field dogs after chronic stress exposure
Source: PeerJ. 2016 Feb 18;4:e1682. doi: 10.7717/peerj.1682 (PMC4768678; doi:10.7717/peerj.1682)
Supplement: Figure S2 [file peerj-04-1682-s002.pdf]

cfa-miR-217 (5'-3')

uugaugucgcagauacugcaucaggaacugauuggauaagaaugggucaccaucaguuccuaaugcauugccuucagcaucu

..((((.(.((.((.(((((.(((((((((.((((........))).).))))))))).))))).)).)).).))))).

Sequence	CFDP1	CFDP2	BP1	BP2	CFDAC1	CFDAC2	BAC1	BAC2	Total reads
	Reads in each sample								
..........gauacugcaucaggaacugauugg......................................	149	415	61	82	48	19	8	43	825
...........Uuacugcaucaggaacugauugg......................................	74	190	23	39	21	6	3	5	361
...........Guacugcaucaggaacugauugga.....................................	22	74	10	10	8	1		6	131
...........Guacugcaucaggaacugauugg......................................	6	30	8	3	1	1	2	3	54
...........Cuacugcaucaggaacugauugg......................................	8	14	1						23
...........Cuacugcaucaggaacugauug.......................................	4	12	2	3	1	1			23
...........auacugUaucaggaacugauu..	3	6	4	6	1				20
...........auacugcaucaggaacugauugUa.....................................	1	12		4					17
...........auacugcaucaggaacugauugU......................................		11	2	1					14
...........auacugcaucaggaacugauuggU.....................................	2	8			1	1			12
...........auacugcaucaggaacugauugga.....................................	5	3		1					9
...........auacugcaucaggaacugauugg......................................	4	4		1					9
...........auacugcaucaggaacugauugA......................................	3	2	1	1					7
...........auacugcaucaggaacugauug.......................................		7							7
...........auacugcaucaggaacugauu..	2	4	1						7
...........auacugcaucaggaacugau...		5		1					6
...........auacugcaucaggaacuga..	2	3					1		6
...........aGacugcaucaggaacugauugg......................................	4	1			1				6
...........aGacugcaucaggaacugauug.......................................		5							5
...........aGacugcaucaggaacugauu..		3	1	1					5
...........aAacugcaucaggaacugauugg......................................	1			2				2	5
............uUcugcaucaggaacugauugga.....................................		3	1						4
............uUcugcaucaggaacugauugg......................................		3				1			4
............uGcugcaucaggaacugauugga.....................................	1	2							3
............uGcugcaucaggaacugauugg......................................						3			3
............uGcugcaucaggaacugauu..		3							3
............uGcugcaucaggaacugau...		3							3
............uacuUcaucaggaacugauugga.....................................		3							3
............uacuUcaucaggaacugauugg......................................		3							3
............uacugcauGaggaacugauugg......................................		2							2
............uacugcaucGggaacugauugga.....................................		2							2
............uacugcaucGggaacugauugg......................................		2							2
............uacugcaucaggaGcugauugga.....................................		2							2
............uacugcaucaggaacugGuugga.....................................		2							2
............uacugcaucaggaacugCuugg......................................		1		1					2
............uacugcaucaggaacugauugU......................................		2							2
............uacugcaucaggaacugauuggU.....................................		2							2
............uacugcaucaggaacugauuggau....................................				1					1
............uacugcaucaggaacugauuggaG....................................		1							1
............uacugcaucaggaacugauuggaAa...................................		1							1
............uacugcaucaggaacugauuggaA....................................		1							1
