# Supplementary material for: Comparison of miRNA expression profiles in pituitary–adrenal axis between Beagle and Chinese Field dogs after chronic stress exposure
Source: PeerJ. 2016 Feb 18;4:e1682. doi: 10.7717/peerj.1682 (PMC4768678; doi:10.7717/peerj.1682)

A: Novel miR-24_20231

Mature Star

5'- cgggcccgggcggcgccuccaggacuguccaaccugagaguguguccccgcggucagucucagguucgucagcccgagaggugccgccccuuccccgcggcggccgcuggc -3'

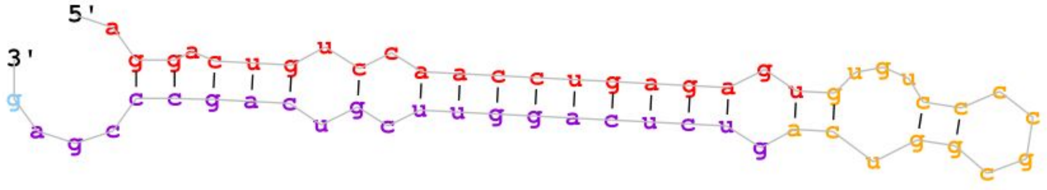

B: Novel miR-21_17074

Mature Star

5'- gaggugaugcgcuggcgcccccaccagcuggcguucccuggcgugggaacgagaggcaggggcugcuggcuggagggagcgccuucucucacucgccgccuccgcguccuc -3'

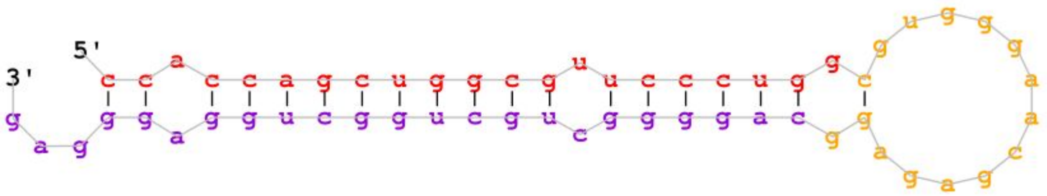

Supplement: Figure S3 [file peerj-04-1682-s003.pdf]
