# Supplementary material for: Comparison of miRNA expression profiles in pituitary–adrenal axis between Beagle and Chinese Field dogs after chronic stress exposure
Source: PeerJ. 2016 Feb 18;4:e1682. doi: 10.7717/peerj.1682 (PMC4768678; doi:10.7717/peerj.1682)
Supplement: Table S2 [file peerj-04-1682-s005.docx]

Table S2. *U6* and *U48*, designed by RIBOBIO (Guangzhou, China), were the reference genes to normalize the cfa-miR-30a，cfa-miR-205, cfa-miR-124 and cfa-miR-222. *GAPDH* was used to normalized the *MMD* expression.

| **MiRNA & gene ID** | **Mature or primer sequence (5'-3')** | **Tm (℃)** |
| --- | --- | --- |
| Cfa-miR-30a | UGUAAACAUCCUCGACUGGAAGC | 60℃ |
| Cfa-miR-205 | UCCUUCAUUCCACCGGAGUCUG | 60℃ |
| Cfa-miR-124 | UAAGGCACGCGGUGAAUGCCA | 60℃ |
| Cfa-miR-222 | AGCUACAUCUGGCUACUGGGU | 60℃ |
| MMD | F:CTCTTCATCGTTTCCACAG | 56.9℃ |
|  | R:GTTCAACCACCTTATACTTTTC |  |
| GAPDH | F:GTCGGAGTGAACGGATTT | 56.9℃ |
|  | R:ATTTGATGTTGGCGGGAT |  |
