# Supplementary material for: Comparison of miRNA expression profiles in pituitary–adrenal axis between Beagle and Chinese Field dogs after chronic stress exposure
Source: PeerJ. 2016 Feb 18;4:e1682. doi: 10.7717/peerj.1682 (PMC4768678; doi:10.7717/peerj.1682)
Supplement: Table S3 [file peerj-04-1682-s006.docx]

Table S3. Total 425 conserved miRNAs identified across 8 samples.

| MiRNAs | CFDAC1 | CFDAC2 | BAC1 | BAC2 | CFDP1 | CFDP2 | BP1 | BP2 |
| --- | --- | --- | --- | --- | --- | --- | --- | --- |
| cfa-let-7a | 1255848.51 | 1230107.75 | 1441115.32 | 1374665.94 | 712721.45 | 598937.47 | 589967.67 | 565082.61 |
| cfa-let-7b | 430212 | 306110.79 | 421360.62 | 358930.64 | 201888.69 | 136414.94 | 123438.57 | 124723.61 |
| cfa-let-7c | 287340.24 | 246987.98 | 307376.25 | 305768.63 | 405235.61 | 383979.97 | 318727.65 | 326200.2 |
| cfa-let-7d | 13421.21242 | 11155.74413 | 10325.17254 | 8683.521072 | 11281.72663 | 7499.67177 | 5549.74274 | 6896.56729 |
| cfa-let-7e | 210990.91 | 216979.91 | 228851.04 | 243593.25 | 194821.29 | 159745.34 | 163231.27 | 171706.21 |
| cfa-let-7f | 777816.91 | 753836.15 | 918074.92 | 884714.22 | 1185280.43 | 1012138.97 | 880298.59 | 899498.92 |
| cfa-let-7g | 3306573.07 | 2869080.6 | 2758636.29 | 2813195.96 | 1938222.26 | 1773452.74 | 1902394.44 | 1849689.83 |
| cfa-let-7j | 14.06835683 | 54.21989856 | 117.3315061 | 37.80923544 | 24.941179 | 11.49668641 | 48.540024 | 10.5775572 |
| cfa-miR-1 | 3357.82 | 1969.41 | 2336.92 | 2638.58 | 1505.72 | 687.74 | 439.33 | 476.67 |
| cfa-miR-101 | 2090208.31 | 1740113.06 | 2700737.95 | 2525644.05 | 1069190.7 | 820301.16 | 1549785.08 | 1434715.82 |
| cfa-miR-103 | 1294498.67 | 1589087.98 | 1385983.13 | 1412133.71 | 1459620.06 | 1287114 | 1579779.38 | 1335758.54 |
| cfa-miR-105a | 0 | 0 | 0 | 0 | 16712.15 | 3166.52 | 2223.28 | 1031.35 |
| cfa-miR-105b | 798.36 | 629.4 | 2074.61 | 1825.89 | 1268.33 | 749.19 | 625.71 | 164.67 |
| cfa-miR-106a | 128066.82 | 110246.18 | 105161.31 | 115168.52 | 38124.58 | 39721.97 | 40791.18 | 39893.18 |
| cfa-miR-106b | 169440.81 | 124742.63 | 160031.19 | 183708.15 | 55108.03 | 60178.5 | 84857.36 | 75730.31 |
| cfa-miR-107 | 1294463.45 | 1589077.83 | 1385959.28 | 1412112.6 | 1459620.06 | 1287114 | 1579766.06 | 1335758.54 |
| cfa-miR-10a | 1242828.15 | 1310274.79 | 1322099.42 | 963554.84 | 18991.07 | 31665.19 | 14591.1 | 9186.79 |
| cfa-miR-10b | 8593051.2 | 10484330.15 | 10830756.36 | 7734338.98 | 69778.63 | 65952.57 | 46782.05 | 48230.62 |
| cfa-miR-1185 | 28333.67066 | 40678.47889 | 51068.53803 | 61868.51225 | 97378.67654 | 100021.1718 | 112596.676 | 85043.5598 |
| cfa-miR-1199-5p | 14.06835683 | 13.55497464 | 0 | 12.60307848 | 8.31372633 | 3.8322288 | 0 | 10.5775572 |
| cfa-miR-122 | 16589.51 | 538.03 | 13067.66 | 591.04 | 88.17 | 49.75 | 13.31 | 34.67 |
| cfa-miR-124 | 8065.81 | 5837.16 | 6199.99 | 24559.86 | 1806444.56 | 67009.05 | 51801.06 | 26407.67 |
| cfa-miR-1249 | 4530.0109 | 4730.686149 | 6247.902699 | 5469.73606 | 19678.59023 | 10665.09276 | 8478.32418 | 7383.13492 |
| cfa-miR-125a | 1361432.02 | 1708957.85 | 1419105.36 | 1424587.78 | 1729022.01 | 1935352.01 | 1108391.02 | 1323945.72 |
| cfa-miR-125b | 2674398.7 | 3404810.02 | 3009473.43 | 3206618.06 | 5966317.3 | 7841984.54 | 4403503.21 | 4879716.86 |
| cfa-miR-126 | 1484896.51 | 1345226.69 | 1493362.13 | 1529888.04 | 539373.64 | 498794.1 | 443337.77 | 416872.04 |
| cfa-miR-127 | 263718.09 | 357355.98 | 438339.04 | 523261.1 | 857745.44 | 847146.34 | 796932.29 | 855185.68 |
| cfa-miR-1271 | 191055.31 | 156557.69 | 130152.02 | 140815.47 | 100245.74 | 149139.55 | 109166.98 | 71492.25 |
| cfa-miR-128 | 62694.98 | 60655.7 | 73875.22 | 80761.51 | 439480.59 | 86511.53 | 105386.08 | 110059.42 |
| cfa-miR-129 | 7220.49 | 8405.51 | 9156.9 | 12475.18 | 204079.44 | 167363.13 | 268896.92 | 293743.12 |
| cfa-miR-1296 | 1857.023102 | 2006.136247 | 1759.972591 | 1651.003281 | 8039.373365 | 4476.043243 | 2993.30148 | 2919.40578 |
| cfa-miR-1301 | 33159.11705 | 31501.76106 | 28511.55598 | 30196.97603 | 25032.62999 | 29347.20818 | 29657.9546 | 18954.9825 |
| cfa-miR-1306 | 4203.15 | 3481.99 | 2956.92 | 2944.65 | 2468.84 | 2379.28 | 2422.97 | 2166.69 |
| cfa-miR-1307 | 410.92 | 456.82 | 238.46 | 274.41 | 874.95 | 1375.48 | 306.2 | 355.34 |
| cfa-miR-130a | 66733.76 | 51559.88 | 68652.93 | 57140.98 | 18258.56 | 19007.9 | 17586.53 | 15071.53 |
| cfa-miR-130b | 1561.5 | 1177.58 | 1550 | 1657.03 | 1790.59 | 1460.34 | 1823.89 | 1542.69 |
| cfa-miR-132 | 64596.96 | 24262.28 | 57087.57 | 35905.73 | 162339.77 | 149133.69 | 98742.87 | 99399.28 |
| cfa-miR-133a | 11811.08 | 6679.74 | 9371.52 | 9140.02 | 5066.55 | 2876.79 | 2050.21 | 1698.69 |
| cfa-miR-133b | 293.52 | 223.33 | 143.08 | 116.1 | 271.3 | 23.41 | 13.31 | 26 |
| cfa-miR-133c | 11811.08 | 6679.74 | 9371.52 | 9140.02 | 5066.55 | 2876.79 | 2050.21 | 1698.69 |
| cfa-miR-134 | 6433.87 | 6527.47 | 8560.75 | 8612.31 | 15545.55 | 13743.04 | 12940.28 | 11968.82 |
| cfa-miR-1343 | 9285.11551 | 8282.089505 | 12261.14239 | 9414.499623 | 22031.37478 | 10113.25181 | 8219.44406 | 9847.70574 |
| cfa-miR-135a-3p | 410.92 | 365.46 | 286.15 | 654.37 | 6497.66 | 12115.89 | 10943.32 | 6890.09 |
| cfa-miR-135a-5p | 39049.34 | 53052.17 | 46833.74 | 71030.44 | 561579.63 | 1131398.51 | 1114634.84 | 688627.54 |
| cfa-miR-135b | 857.07 | 994.85 | 1406.92 | 1540.93 | 10065.27 | 21000.87 | 28942.56 | 15071.53 |
| cfa-miR-136 | 46481.16 | 56899.61 | 83413.66 | 88160.07 | 149507.23 | 138226.47 | 138788.51 | 158914.05 |
| cfa-miR-137 | 36419.44 | 50463.51 | 50243.74 | 69837.8 | 87670.94 | 63707.91 | 85895.78 | 76683.65 |
| cfa-miR-138a | 6527.79 | 7958.84 | 10468.44 | 35789.63 | 52944.4 | 7942.64 | 10264.36 | 8155.44 |
| cfa-miR-138b | 598.77 | 984.7 | 1216.15 | 4242.83 | 8715.55 | 1413.52 | 1570.94 | 1066.01 |
| cfa-miR-139 | 7373.12 | 6212.77 | 5627.68 | 5319.37 | 4883.42 | 1123.79 | 1650.82 | 615.34 |
| cfa-miR-140 | 383284.69 | 350666.08 | 396226.83 | 348977.94 | 63830.36 | 80678.93 | 89104.22 | 55068.71 |
| cfa-miR-141 | 4531.88 | 3512.45 | 2384.61 | 2469.71 | 2315039.09 | 3512334.22 | 4626709.76 | 3372330.11 |
| cfa-miR-142 | 241563.52 | 220725.84 | 160269.65 | 229376.61 | 163126.55 | 86362.28 | 67510.46 | 65113.51 |
| cfa-miR-143 | 10111291.11 | 7168041.93 | 9142690.81 | 9274211.39 | 869255.38 | 1310915.58 | 1192556.1 | 835668.1 |
| cfa-miR-144 | 989606.18 | 840814.9 | 877155.01 | 1411331.58 | 162115.95 | 341015.4 | 561344.62 | 449779.79 |
| cfa-miR-145 | 3740812.11 | 2661095 | 2467809.23 | 2956808.32 | 309574.86 | 465531.01 | 371607.07 | 281566.29 |
| cfa-miR-1468 | 1899.228172 | 2358.565587 | 2346.630122 | 1613.194045 | 1596.235456 | 1728.335191 | 1181.14058 | 1427.97022 |
| cfa-miR-146a | 378330.15 | 437259.07 | 324521.6 | 343553.03 | 455968.91 | 413207.36 | 256728.79 | 181491 |
| cfa-miR-146b | 109058.74 | 103028.4 | 77881.37 | 71800.9 | 169549.6 | 163222.07 | 109353.37 | 51298.66 |
| cfa-miR-147 | 46.96 | 10.15 | 47.69 | 42.22 | 81.39 | 172.67 | 119.82 | 277.34 |
| cfa-miR-148a | 1534242.39 | 1776201.93 | 2317507.25 | 2442486.72 | 2048044.93 | 1881415.83 | 2807002.65 | 3022651.6 |
| cfa-miR-148b | 672691.29 | 675242.6 | 724229.96 | 643886.2 | 543219.33 | 526941.59 | 853592.62 | 640249.58 |
| cfa-miR-149 | 12621.18 | 11532.2 | 10110.75 | 15514.82 | 43659.12 | 26169.15 | 17386.84 | 16614.21 |
| cfa-miR-150 | 9650.8 | 11806.29 | 6223.83 | 10269.33 | 17024.14 | 4521.51 | 3980.6 | 4775.39 |
| cfa-miR-151 | 663768.41 | 693007.87 | 639981.68 | 607579.4 | 701299.68 | 808357.94 | 594081.4 | 546726.38 |
| cfa-miR-152 | 2003010.63 | 1320426.37 | 1847262.12 | 1582902.29 | 296016.59 | 290819.34 | 366388.36 | 308944.65 |
| cfa-miR-153 | 144914.63 | 138203.63 | 213971.07 | 225028.24 | 380886.34 | 458021.51 | 524627.23 | 444631.73 |
| cfa-miR-155 | 33742.58 | 37936.46 | 37366.84 | 35145.82 | 26499.33 | 14735.14 | 12913.66 | 7254.09 |
| cfa-miR-15a | 172822.11 | 147604 | 193320.35 | 162346.25 | 62799.41 | 58124.07 | 61160.14 | 70460.91 |
| cfa-miR-15b | 76231.93 | 79009.76 | 70584.46 | 65827.17 | 50380.61 | 62452.42 | 40937.62 | 43793.23 |
| cfa-miR-16 | 1200526.65 | 1109141.5 | 1190945.86 | 1006246.99 | 564428.29 | 532911.74 | 442858.5 | 504805.17 |
| cfa-miR-17 | 7913.19 | 7167.02 | 7702.29 | 7187.48 | 2970.75 | 3189.93 | 3141.88 | 3328.04 |
| cfa-miR-181a | 1064065.28 | 1152894.82 | 946523.32 | 1001571.43 | 2144383.94 | 2085170.51 | 1600920.5 | 1297382.05 |
| cfa-miR-181b | 101873.47 | 90156.2 | 79312.13 | 90270.93 | 170546.63 | 152935.27 | 98676.3 | 92881.86 |
| cfa-miR-181c | 115880.04 | 130569.64 | 149443.52 | 152520.19 | 482068.07 | 487541.54 | 447944.08 | 486856.27 |
| cfa-miR-181d | 30067.76 | 32789.61 | 31786.85 | 42438.84 | 152132.07 | 163833.72 | 133782.81 | 143937.85 |
| cfa-miR-182 | 28975.88 | 29937.01 | 22939.95 | 15409.28 | 562474.93 | 602147.88 | 550960.44 | 599602.39 |
| cfa-miR-183 | 48582.74 | 47113.49 | 35983.77 | 27778.92 | 910031.93 | 1686212.5 | 1789539.74 | 1079464.57 |
| cfa-miR-1835 | 82.18 | 111.67 | 47.69 | 94.99 | 196.69 | 79.02 | 26.63 | 17.33 |
| cfa-miR-1836 | 0 | 60.91 | 0 | 10.55 | 0 | 64.38 | 0 | 26 |
| cfa-miR-1837 | 11.74 | 0 | 0 | 0 | 0 | 0 | 0 | 0 |
| cfa-miR-1838 | 28952.4 | 34982.35 | 35816.84 | 34913.63 | 13504.01 | 18369.91 | 16947.51 | 14126.85 |
| cfa-miR-1839 | 93.93 | 131.97 | 23.85 | 63.33 | 128.87 | 70.24 | 53.25 | 43.33 |
| cfa-miR-184 | 211.33 | 101.52 | 166.92 | 137.21 | 447.65 | 699.44 | 252.95 | 442.01 |
| cfa-miR-1840 | 58.7 | 20.3 | 47.69 | 0 | 6.78 | 32.19 | 13.31 | 26 |
| cfa-miR-1841 | 258.29 | 152.27 | 166.92 | 189.98 | 162.78 | 99.5 | 119.82 | 69.33 |
| cfa-miR-1842 | 6480.83 | 7106.11 | 5675.37 | 8453.99 | 8498.51 | 7875.33 | 8613.54 | 8146.77 |
| cfa-miR-1843 | 0 | 0 | 0 | 0 | 20.35 | 5.85 | 13.31 | 0 |
| cfa-miR-1844 | 129.15 | 91.36 | 143.08 | 168.87 | 156 | 178.52 | 173.07 | 95.33 |
| cfa-miR-185 | 187908.83 | 219527.95 | 163465.03 | 130282.28 | 259662.25 | 202209.47 | 136432.1 | 144995.2 |
| cfa-miR-186 | 3402541 | 3581630.27 | 2984768.87 | 2481664.28 | 1541851.41 | 1533400.14 | 1386553.82 | 1337439.9 |
| cfa-miR-187 | 180829.22 | 139005.61 | 139857.39 | 156752.47 | 2136.5 | 1445.71 | 2396.35 | 1109.35 |
| cfa-miR-188 | 5788.13 | 4476.85 | 6557.68 | 5340.48 | 3852.48 | 4439.57 | 4566.38 | 4732.06 |
| cfa-miR-18a | 10543.09 | 9481.58 | 9729.21 | 12559.62 | 3398.05 | 3181.15 | 5045.64 | 4645.39 |
| cfa-miR-18b | 974.47 | 680.16 | 882.31 | 1150.42 | 637.56 | 617.5 | 852.03 | 979.35 |
| cfa-miR-190a | 58808.83 | 56483.4 | 75282.14 | 84307.75 | 27136.89 | 38931.8 | 55994.67 | 28609.04 |
| cfa-miR-190b | 2218.98 | 3390.63 | 3481.53 | 3387.93 | 11625.25 | 8721.1 | 7388.74 | 9056.78 |
| cfa-miR-191 | 1208745.09 | 1226950.61 | 929664.12 | 797651.8 | 1505286.81 | 1622674.33 | 922687.33 | 946689.53 |
| cfa-miR-192 | 95545.27 | 61488.13 | 73589.07 | 65795.51 | 105088.47 | 90386.27 | 114958.16 | 92812.53 |
| cfa-miR-193a | 6656.94 | 4517.45 | 5484.6 | 6205.93 | 3669.35 | 3040.68 | 2303.16 | 2669.37 |
| cfa-miR-193b | 1925.46 | 1725.77 | 1526.15 | 1129.31 | 569.73 | 579.46 | 372.77 | 355.34 |
| cfa-miR-194 | 82783.2 | 55356.57 | 67722.93 | 52824.27 | 74160.15 | 63380.14 | 80876.76 | 66006.18 |
| cfa-miR-195 | 305209.49 | 232643.8 | 312837.01 | 340661.15 | 97057.95 | 123625.95 | 112694.94 | 96088.57 |
| cfa-miR-196a | 399.18 | 1340.01 | 858.46 | 84.43 | 0 | 0 | 0 | 0 |
| cfa-miR-196b | 2254.2 | 4344.88 | 10229.98 | 1931.44 | 20.35 | 29.27 | 26.63 | 17.33 |
| cfa-miR-197 | 91107.31 | 79679.76 | 92952.1 | 86492.49 | 34889.32 | 37193.43 | 45144.54 | 36807.81 |
| cfa-miR-199 | 909488.11 | 750161.27 | 957945.6 | 1231159.12 | 85636.18 | 128165.01 | 117780.52 | 119289.54 |
| cfa-miR-19a | 729891.65 | 844966.9 | 843293.54 | 628519.13 | 252113.29 | 168343.52 | 280119.82 | 266832.77 |
| cfa-miR-19b | 269353.59 | 299684.84 | 339330.03 | 291699.75 | 96610.31 | 66043.29 | 102457.21 | 126725.63 |
| cfa-miR-200a | 58.7 | 81.21 | 47.69 | 52.77 | 15986.42 | 16491.07 | 15030.43 | 16232.88 |
| cfa-miR-200b | 129.15 | 30.45 | 23.85 | 42.22 | 20869.83 | 21246.7 | 16801.06 | 19075.58 |
| cfa-miR-200c | 1385.39 | 1340.01 | 977.69 | 823.24 | 1509376.68 | 1617956.74 | 1276880.94 | 1469157.59 |
| cfa-miR-202 | 7718714.55 | 7747423.14 | 7427822.25 | 6877350.91 | 7365.82 | 1597.89 | 1397.87 | 1291.35 |
| cfa-miR-203 | 1120643.38 | 1360809.36 | 1146592.11 | 982162.07 | 79077.48 | 79821.45 | 95041.84 | 90862.5 |
| cfa-miR-204 | 30408.24 | 19978.31 | 22129.18 | 30649.69 | 148259.25 | 165089.21 | 118978.7 | 122244.91 |
| cfa-miR-205 | 93.93 | 172.58 | 47.69 | 116.1 | 12764.71 | 55847.22 | 10730.32 | 22724.29 |
| cfa-miR-206 | 1502.8 | 1390.77 | 2217.69 | 707.14 | 746.08 | 2.93 | 13.31 | 17.33 |
| cfa-miR-207 | 0 | 0 | 29.33287652 | 0 | 8.31372633 | 0 | 0 | 0 |
| cfa-miR-208a | 23.48 | 20.3 | 23.85 | 10.55 | 5582.02 | 10187.3 | 8360.59 | 6023.41 |
| cfa-miR-208b | 82.18 | 182.73 | 286.15 | 200.53 | 223.82 | 336.55 | 559.15 | 286 |
| cfa-miR-20a | 371884.54 | 361782.07 | 357381.53 | 360376.58 | 80284.77 | 80828.19 | 102111.07 | 101279.97 |
| cfa-miR-20b | 2759.05 | 3421.08 | 2003.07 | 3197.95 | 3520.13 | 2932.4 | 2236.59 | 3076.71 |
| cfa-miR-21 | 11362478.59 | 8917961.41 | 8270829.64 | 9166958.59 | 2339781.75 | 1411951.54 | 1716411.18 | 1605078.68 |
| cfa-miR-210 | 4461.44 | 3806.84 | 6605.37 | 6279.81 | 3479.44 | 3345.04 | 5817.8 | 5243.4 |
| cfa-miR-211 | 0 | 0 | 238.46 | 221.64 | 1526.07 | 1685.69 | 1397.87 | 1300.02 |
| cfa-miR-2114 | 3967.276627 | 3198.974015 | 4487.930108 | 3654.892759 | 1837.33352 | 3084.944187 | 3575.78176 | 2284.75235 |
| cfa-miR-212 | 14534.9 | 8080.66 | 14355.35 | 10121.57 | 43876.16 | 67044.17 | 37915.56 | 31720.41 |
| cfa-miR-214 | 48254 | 36545.69 | 43280.67 | 47779.32 | 3452.31 | 5270.7 | 3275.01 | 3241.38 |
| cfa-miR-215 | 8688.07 | 5370.19 | 7392.29 | 5699.32 | 596.86 | 1940.3 | 1144.92 | 754.01 |
| cfa-miR-216a | 223.07 | 142.12 | 166.92 | 263.86 | 1519.29 | 2718.76 | 918.6 | 762.68 |
| cfa-miR-216b | 751.4 | 203.03 | 214.61 | 517.16 | 3893.17 | 5815.04 | 2902.24 | 2080.03 |
| cfa-miR-217 | 962.73 | 355.31 | 333.85 | 622.7 | 1987.28 | 2531.46 | 1544.31 | 1378.02 |
| cfa-miR-218 | 339550.84 | 269087.97 | 406027.58 | 588022.28 | 436421.67 | 293631.74 | 376200.07 | 352833.21 |
| cfa-miR-219-3p | 35.22 | 10.15 | 0 | 0 | 10445.09 | 2774.36 | 1118.3 | 424.67 |
| cfa-miR-219-5p | 8077.56 | 8273.54 | 9562.29 | 9741.62 | 4422.21 | 2563.65 | 3661.09 | 3250.04 |
| cfa-miR-22 | 2820006.03 | 2040488.21 | 2290489.62 | 2258852.45 | 843264.74 | 781108.9 | 518543.17 | 635170.85 |
| cfa-miR-221 | 50942.6 | 36606.6 | 22558.41 | 33837.09 | 90709.51 | 19203.97 | 10703.69 | 10738.14 |
| cfa-miR-222 | 94946.5 | 59457.81 | 37128.38 | 50882.28 | 153481.79 | 29888.78 | 17160.52 | 19378.92 |
| cfa-miR-223 | 10543.09 | 7867.48 | 4745.37 | 6849.74 | 2428.14 | 3002.63 | 1624.19 | 1898.02 |
| cfa-miR-224 | 3545.67 | 2649.56 | 3123.84 | 3261.28 | 2231.45 | 1875.91 | 625.71 | 1378.02 |
| cfa-miR-2387 | 633.0760575 | 1003.068123 | 821.3205426 | 529.3292961 | 881.2549914 | 1651.690615 | 1618.0008 | 1089.48839 |
| cfa-miR-23a | 36243.33 | 35530.54 | 32812.24 | 42871.57 | 17566.74 | 14705.88 | 11728.79 | 9984.13 |
| cfa-miR-23b | 20593.07 | 17633.3 | 18599.96 | 23905.49 | 9787.19 | 10160.96 | 9465.58 | 7453.43 |
| cfa-miR-24 | 2080217.03 | 2035422.57 | 2290799.62 | 2631377.03 | 1868057.03 | 2044620.34 | 1717835.67 | 1667696.15 |
| cfa-miR-2483 | 2265.00545 | 3131.199142 | 3519.945183 | 4385.87131 | 4289.882788 | 6794.54167 | 7604.60375 | 3522.32654 |
| cfa-miR-25 | 177154.41 | 159379.83 | 163417.34 | 166610.18 | 96637.44 | 123573.27 | 101991.25 | 99529.28 |
| cfa-miR-26a | 9605727.73 | 9394090.89 | 9373878.77 | 8741314.22 | 7900619.26 | 7717515.74 | 6396173.42 | 6323870.8 |
| cfa-miR-26b | 2753612.75 | 2877932.78 | 2535794.47 | 2246271.72 | 1311170.9 | 1510271.67 | 1109003.42 | 1117598.4 |
| cfa-miR-27a | 365239.34 | 345955.75 | 300484.73 | 383226.64 | 240481.26 | 254465.82 | 152274.63 | 177105.61 |
| cfa-miR-27b | 5225156.81 | 4532650.73 | 5122166.51 | 5830649.29 | 3322590.19 | 4124008 | 3439612.01 | 3324948.83 |
| cfa-miR-28 | 68095.67 | 66584.22 | 69058.31 | 57024.88 | 18156.82 | 24869.76 | 20209.2 | 18486.24 |
| cfa-miR-299 | 4050.52 | 5715.34 | 6939.22 | 7926.28 | 17275.1 | 15952.58 | 12620.77 | 14187.52 |
| cfa-miR-29a | 3936458.01 | 3129052.45 | 3368500.34 | 3569168.82 | 4012135.75 | 3820759.64 | 4014629.12 | 4175757.79 |
| cfa-miR-29b | 322010.33 | 263636.57 | 287059.37 | 280459.42 | 357486.63 | 341928.48 | 279747.05 | 285830.35 |
| cfa-miR-29c | 709122.47 | 485651.66 | 697617.71 | 749566.4 | 304406.57 | 278293.73 | 346485.35 | 359315.96 |
| cfa-miR-301a | 10918.79 | 11654.02 | 12972.28 | 14438.28 | 15355.64 | 12434.88 | 15083.68 | 16198.21 |
| cfa-miR-301b | 904.03 | 558.34 | 1049.23 | 1255.96 | 2055.11 | 1480.83 | 2343.1 | 2201.36 |
| cfa-miR-302a | 0 | 0 | 0 | 0 | 0 | 0 | 0 | 8.67 |
| cfa-miR-302b | 0 | 0 | 0 | 37.80923544 | 0 | 3.8322288 | 16.180008 | 0 |
| cfa-miR-302c | 14.06835683 | 0 | 0 | 0 | 0 | 0 | 0 | 0 |
| cfa-miR-302d | 28.13671367 | 13.55497464 | 0 | 0 | 0 | 0 | 0 | 0 |
| cfa-miR-30a | 5489755.45 | 4844690.05 | 5658608.42 | 4963782.4 | 2553207.51 | 5289957.28 | 6285675.13 | 4436272.48 |
| cfa-miR-30b | 2618783.33 | 2307718.44 | 2432731.61 | 1863287.83 | 1090169.06 | 1126397.05 | 962147.2 | 1027264.57 |
| cfa-miR-30c | 2397460.66 | 2274979.59 | 2359142.54 | 2076643.01 | 964421.01 | 1287915.88 | 1240190.18 | 1049789.52 |
| cfa-miR-30d | 5056338.25 | 5151399.78 | 4390377.34 | 3785721.96 | 3332492.68 | 3599249.62 | 2632734.87 | 3220912.82 |
| cfa-miR-30e | 72721.48 | 84816.46 | 83509.05 | 67241.45 | 33437.86 | 46552.52 | 34534.04 | 32249.08 |
| cfa-miR-31 | 1890.24 | 2243.5 | 786.92 | 2290.28 | 91055.42 | 79081.04 | 75072.27 | 96885.91 |
| cfa-miR-32 | 72815.4 | 63914.36 | 71132.92 | 74376.15 | 34251.76 | 37925.07 | 44066.18 | 45595.92 |
| cfa-miR-320 | 182801.65 | 162709.55 | 177248.07 | 171560.15 | 66807.89 | 86789.55 | 74872.57 | 68528.22 |
| cfa-miR-323 | 15791.15 | 19084.97 | 26636.1 | 31399.04 | 35452.27 | 35490.18 | 35505.9 | 30455.06 |
| cfa-miR-324 | 4144.44 | 3299.26 | 3696.15 | 3081.86 | 6965.65 | 5501.9 | 4792.7 | 4749.39 |
| cfa-miR-325 | 117.41 | 162.43 | 214.61 | 263.86 | 2428.14 | 2148.08 | 2170.03 | 1993.36 |
| cfa-miR-326 | 16753.88 | 11166.74 | 15976.89 | 14216.64 | 13307.32 | 4126.43 | 5618.11 | 2955.37 |
| cfa-miR-328 | 4848.88 | 4487 | 5007.68 | 4833.87 | 7338.69 | 5458 | 3567.9 | 4766.73 |
| cfa-miR-329a | 0 | 0 | 0 | 10.55 | 101.74 | 46.82 | 106.5 | 26 |
| cfa-miR-329b | 8605.88 | 9816.58 | 11779.97 | 16095.31 | 22077.12 | 22733.38 | 23430.96 | 22940.96 |
| cfa-miR-330 | 2841.23 | 2507.44 | 3266.92 | 3219.06 | 9454.84 | 3792.8 | 3953.97 | 2842.7 |
| cfa-miR-331 | 45130.99 | 42453.91 | 37843.76 | 46945.53 | 58845.2 | 64901.94 | 47554.2 | 56403.39 |
| cfa-miR-335 | 925244.04 | 1236026.12 | 1054927.7 | 1179939.11 | 4575892.58 | 4631148.6 | 4001569.02 | 4075890.5 |
| cfa-miR-338 | 75504.01 | 79629.01 | 62452.94 | 168721.04 | 72403.47 | 44743.91 | 37422.97 | 27829.03 |
| cfa-miR-339 | 8863.064805 | 6140.403512 | 7333.219131 | 7511.434773 | 3300.549354 | 2533.10324 | 3106.56153 | 2623.23418 |
| cfa-miR-33a | 5952.5 | 4832.15 | 4959.99 | 7894.62 | 5500.63 | 4550.77 | 5671.36 | 6257.41 |
| cfa-miR-33b | 598.77 | 436.52 | 214.61 | 443.28 | 312 | 292.65 | 226.32 | 251.34 |
| cfa-miR-340 | 148155.04 | 222867.82 | 287917.83 | 339510.73 | 220113.33 | 170409.66 | 327141.5 | 284738.33 |
| cfa-miR-342 | 170027.84 | 162333.94 | 137782.78 | 158873.88 | 381293.29 | 466757.24 | 365523.01 | 240728.43 |
| cfa-miR-345 | 3299.12 | 2070.92 | 2026.92 | 1678.13 | 1824.5 | 1264.27 | 878.66 | 1161.35 |
| cfa-miR-346 | 0 | 0 | 0 | 0 | 1329.38 | 234.12 | 199.7 | 121.33 |
| cfa-miR-34a | 197301.33 | 172140.37 | 84319.82 | 60813.88 | 9678.67 | 7284.16 | 6949.41 | 5668.07 |
| cfa-miR-34b | 0 | 0 | 0 | 0 | 115.3 | 269.24 | 1331.3 | 1906.69 |
| cfa-miR-34c | 270.03 | 294.4 | 667.69 | 717.69 | 6619.75 | 12721.68 | 53531.76 | 77871 |
| cfa-miR-350 | 8171.48 | 8486.72 | 11589.21 | 10733.72 | 5812.63 | 5027.8 | 5511.6 | 5130.73 |
| cfa-miR-361 | 188178.86 | 187245.92 | 160365.03 | 145480.47 | 206432.98 | 178808.84 | 108607.84 | 121404.23 |
| cfa-miR-362 | 242350.14 | 200219.64 | 158385.81 | 162515.12 | 271640.19 | 367556.21 | 266234.31 | 228828.95 |
| cfa-miR-363 | 21637.99 | 21744.69 | 17336.12 | 27557.28 | 53934.65 | 50620.41 | 46755.42 | 63172.15 |
| cfa-miR-365 | 88277.82 | 74634.43 | 90829.8 | 75009.41 | 14487.48 | 15373.13 | 13006.85 | 15166.86 |
| cfa-miR-369 | 77405.99 | 116814.25 | 141526.61 | 168816.03 | 351253.48 | 291600.72 | 341586.15 | 400942.5 |
| cfa-miR-370 | 23082.08 | 28759.43 | 28829.94 | 34786.97 | 135562.36 | 130883.77 | 93697.22 | 95889.24 |
| cfa-miR-371 | 82.18 | 111.67 | 23.85 | 94.99 | 61.04 | 5.85 | 26.63 | 0 |
| cfa-miR-374a | 8402230.7 | 7377763.45 | 6606705.58 | 8459683.27 | 2786702.77 | 4550098 | 7711742.07 | 3956626.97 |
| cfa-miR-374b | 230280.77 | 257708.05 | 243993.31 | 245545.8 | 88857.88 | 156318.36 | 157972.61 | 111186.1 |
| cfa-miR-375 | 1418456.27 | 1878895.33 | 2153279.15 | 2164307.03 | 10726742.08 | 12287973.7 | 9585912.85 | 11114510.5 |
| cfa-miR-376a | 10637.01 | 11115.98 | 12924.59 | 15578.15 | 35269.14 | 33403.56 | 30367.06 | 33575.1 |
| cfa-miR-376b | 1866.76 | 2324.71 | 3076.15 | 3620.12 | 4130.56 | 3789.87 | 3874.1 | 4437.39 |
| cfa-miR-376c | 1819.8 | 1959.26 | 3147.69 | 3831.21 | 5582.02 | 4980.98 | 4912.51 | 5754.74 |
| cfa-miR-377 | 504.85 | 680.16 | 500.77 | 971 | 3411.61 | 2891.42 | 1810.57 | 2123.36 |
| cfa-miR-378 | 277677.7 | 154689.8 | 191555.74 | 168963.79 | 61429.34 | 62230.01 | 40138.84 | 32188.41 |
| cfa-miR-379 | 64456.07 | 84836.77 | 136590.47 | 150683.74 | 283136.57 | 252809.4 | 296601.37 | 297010.49 |
| cfa-miR-380 | 16777.36 | 18354.06 | 26659.94 | 34238.15 | 61225.87 | 54354.68 | 61106.88 | 67626.87 |
| cfa-miR-381 | 135498.64 | 145421.41 | 203264.17 | 245577.46 | 429985.05 | 387339.64 | 451352.22 | 494751.71 |
| cfa-miR-382 | 8218.44 | 10638.86 | 14069.2 | 21298.58 | 25902.47 | 21694.46 | 28583.11 | 31711.74 |
| cfa-miR-383 | 93.93 | 223.33 | 286.15 | 348.29 | 24389.97 | 16014.04 | 8134.27 | 13632.84 |
| cfa-miR-384 | 1960.69 | 2162.29 | 2718.46 | 3810.1 | 23874.49 | 24278.6 | 21114.49 | 19136.25 |
| cfa-miR-3958 | 22228.0038 | 32437.05431 | 43969.98191 | 56739.05931 | 121347.1496 | 124838.6855 | 112014.195 | 116300.241 |
| cfa-miR-409 | 18561.94 | 25856.08 | 0 | 0 | 80745.98 | 86180.83 | 72476.23 | 78798.35 |
| cfa-miR-410 | 61544.4 | 77547.93 | 106830.54 | 143665.14 | 329637.57 | 256379.78 | 323600.23 | 332830.29 |
| cfa-miR-411 | 413505.08 | 558793.81 | 873005.79 | 896693.35 | 1849683.16 | 1745117.95 | 1911780.14 | 1837131.67 |
| cfa-miR-421 | 1009.69 | 1055.76 | 1406.92 | 1308.73 | 2414.58 | 1911.03 | 1424.5 | 1690.02 |
| cfa-miR-423a | 97541.18 | 105261.75 | 91092.11 | 82144.12 | 59781.19 | 59947.3 | 39273.49 | 45959.93 |
| cfa-miR-424 | 3803.96 | 5106.25 | 2241.53 | 2860.22 | 40.7 | 70.24 | 79.88 | 34.67 |
| cfa-miR-425 | 226641.17 | 220949.17 | 179155.76 | 141364.3 | 164835.74 | 118612.78 | 120536.33 | 99147.94 |
| cfa-miR-429 | 763.14 | 578.64 | 620 | 791.57 | 174785.71 | 213693.23 | 191641.31 | 206390.66 |
| cfa-miR-432 | 11752.37 | 17095.26 | 20913.03 | 24042.7 | 55949.06 | 46631.53 | 40697.98 | 42233.21 |
| cfa-miR-433 | 5107.18 | 7776.11 | 8322.29 | 12295.76 | 34197.5 | 29452.73 | 20648.54 | 24691.65 |
| cfa-miR-448 | 11.74 | 0 | 0 | 21.11 | 2645.19 | 918.93 | 1903.77 | 693.34 |
| cfa-miR-449a | 126.6152115 | 162.6596957 | 87.99862957 | 176.4430987 | 1479.843287 | 1402.595742 | 1828.3409 | 4749.32318 |
| cfa-miR-449b | 0 | 13.55497464 | 0 | 0 | 0 | 11.49668641 | 0 | 63.4653431 |
| cfa-miR-450a | 1816851.16 | 2767219.63 | 1472210.63 | 1397410.46 | 23318.33 | 20892.59 | 26106.88 | 26919.01 |
| cfa-miR-450b | 1884723.75 | 2588064.52 | 1475382.16 | 1512389.01 | 14514.61 | 12361.72 | 17719.67 | 17463.56 |
| cfa-miR-451 | 2376996.74 | 1983212.98 | 1143897.5 | 1833566.92 | 682661.3 | 1001351.73 | 749844.04 | 792334.21 |
| cfa-miR-452 | 61908.36 | 62960.11 | 78167.52 | 89827.65 | 93517.48 | 78644.98 | 70213.01 | 106194.03 |
| cfa-miR-454 | 10190.87 | 10435.83 | 7630.75 | 10649.29 | 16637.54 | 12036.87 | 9931.53 | 12462.83 |
| cfa-miR-455 | 18667.61 | 17481.02 | 20197.65 | 23588.86 | 3337 | 4430.79 | 8640.17 | 5711.41 |
| cfa-miR-483 | 0 | 0 | 453.08 | 453.83 | 0 | 0 | 0 | 0 |
| cfa-miR-485 | 6410.39 | 9948.55 | 10301.52 | 10870.93 | 43367.47 | 33166.51 | 23497.53 | 26321.01 |
| cfa-miR-486 | 249389.7616 | 169572.7327 | 155698.9086 | 231846.2317 | 53224.47599 | 71911.77351 | 93099.7659 | 79522.0749 |
| cfa-miR-486-3p | 365.7772777 | 420.2042138 | 381.3273948 | 390.6954328 | 340.8627797 | 433.0418549 | 679.560335 | 370.214502 |
| cfa-miR-487a | 32744.62 | 49509.26 | 49671.43 | 61679.33 | 147316.48 | 142531.41 | 114412.32 | 112234.78 |
| cfa-miR-487b | 32462.85 | 46880 | 53916.04 | 66967.04 | 166239.73 | 170196.02 | 155416.51 | 173404.9 |
| cfa-miR-488 | 551.81 | 670 | 786.92 | 1467.05 | 4686.73 | 3406.5 | 4273.49 | 3484.04 |
| cfa-miR-489 | 46.96 | 20.3 | 119.23 | 73.88 | 128.87 | 105.36 | 119.82 | 34.67 |
| cfa-miR-490 | 493.11 | 487.28 | 1096.92 | 2406.38 | 2916.49 | 1436.93 | 1491.06 | 901.34 |
| cfa-miR-491 | 410.92 | 172.58 | 238.46 | 401.06 | 257.74 | 158.03 | 119.82 | 104 |
| cfa-miR-493 | 187.85 | 294.4 | 405.38 | 833.79 | 481.56 | 901.38 | 572.46 | 598.01 |
| cfa-miR-494 | 54124.32 | 73994.88 | 83365.97 | 88233.95 | 207158.71 | 212440.67 | 171485.35 | 188225.09 |
| cfa-miR-495 | 7725.34 | 11978.87 | 12471.51 | 16221.96 | 87982.93 | 63087.48 | 55981.36 | 67314.87 |
| cfa-miR-496 | 504.85 | 923.79 | 977.69 | 2068.64 | 5758.37 | 7047.12 | 3780.91 | 4489.39 |
| cfa-miR-497 | 107779.01 | 87313.75 | 119850.51 | 138345.77 | 30060.16 | 34085.44 | 49604.41 | 41236.53 |
| cfa-miR-499 | 389131.53 | 372552.89 | 372189.96 | 482173.21 | 72566.25 | 56760.3 | 96958.92 | 94762.55 |
| cfa-miR-500 | 67320.79 | 56026.58 | 54822.19 | 55895.57 | 63626.88 | 73786.92 | 62038.8 | 60979.45 |
| cfa-miR-502 | 72275.33 | 55630.67 | 54178.34 | 55557.84 | 69812.55 | 69405.89 | 54889.69 | 59358.76 |
| cfa-miR-503 | 180312.64 | 207802.88 | 114628.21 | 109469.2 | 1587.11 | 1176.47 | 1570.94 | 1040.01 |
| cfa-miR-504 | 2207.24 | 1949.1 | 1788.46 | 2934.1 | 14758.78 | 13640.62 | 9478.89 | 6144.75 |
| cfa-miR-505 | 1197.54 | 1045.61 | 1502.3 | 1192.64 | 963.12 | 880.89 | 812.1 | 944.68 |
| cfa-miR-506 | 168.820282 | 230.4345689 | 58.66575305 | 163.8400202 | 307.6078743 | 249.0948723 | 161.80008 | 359.636944 |
| cfa-miR-507a | 886.3064805 | 623.5288334 | 263.9958887 | 642.7570024 | 2344.470826 | 2303.169511 | 2977.12147 | 3638.67967 |
| cfa-miR-507b | 267.2987798 | 311.7644167 | 58.66575305 | 289.870805 | 249.41179 | 195.443669 | 258.880128 | 497.145188 |
| cfa-miR-508a | 70.34178416 | 54.21989856 | 87.99862957 | 75.61847087 | 224.470611 | 145.6246946 | 80.9000399 | 137.508243 |
| cfa-miR-508b | 562.7342733 | 379.5392899 | 205.3301357 | 604.947767 | 640.1569277 | 521.1831174 | 647.200319 | 973.135261 |
| cfa-miR-514 | 11.74 | 71.06 | 0 | 31.66 | 189.91 | 70.24 | 159.76 | 164.67 |
| cfa-miR-532 | 57822.62 | 47103.34 | 57826.8 | 55072.34 | 42472.18 | 44111.78 | 45384.18 | 52746.01 |
| cfa-miR-539 | 3968.33 | 5045.34 | 4149.22 | 5129.39 | 23453.98 | 25036.57 | 13832.26 | 13632.84 |
| cfa-miR-542 | 684361.48 | 820684.31 | 628821.7 | 595790.25 | 5575.24 | 4840.5 | 9532.14 | 8190.11 |
| cfa-miR-543 | 2218.98 | 3563.21 | 2098.46 | 3683.45 | 22653.64 | 18484.04 | 13326.36 | 14404.19 |
| cfa-miR-544 | 528.33 | 822.28 | 1001.54 | 1509.26 | 1444.68 | 1144.28 | 1424.5 | 1516.69 |
| cfa-miR-545 | 35.22 | 40.61 | 47.69 | 73.88 | 0 | 0 | 0 | 0 |
| cfa-miR-551a | 1843.28 | 1136.98 | 1168.46 | 1688.69 | 847.82 | 482.88 | 492.58 | 372.67 |
| cfa-miR-551b | 35.22 | 30.45 | 119.23 | 31.66 | 3682.91 | 1814.46 | 4300.11 | 3432.04 |
| cfa-miR-574 | 93197.14 | 60239.49 | 74805.22 | 84012.23 | 148530.55 | 136420.79 | 109566.37 | 124844.94 |
| cfa-miR-578 | 0 | 13.55497464 | 0 | 12.60307848 | 8.31372633 | 0 | 0 | 0 |
| cfa-miR-582 | 4109.22 | 3197.75 | 3338.45 | 3567.35 | 6138.19 | 3994.73 | 3155.19 | 2894.7 |
| cfa-miR-589 | 0 | 0 | 29.33287652 | 12.60307848 | 8.31372633 | 0 | 0 | 0 |
| cfa-miR-590 | 181240.15 | 162821.22 | 178345 | 179613.08 | 58824.85 | 56921.26 | 74619.63 | 66283.52 |
| cfa-miR-592 | 2031.13 | 4893.06 | 4316.14 | 6786.42 | 2238.23 | 992.1 | 1211.49 | 910.01 |
| cfa-miR-599 | 0 | 10.15 | 0 | 0 | 0 | 0 | 0 | 0 |
| cfa-miR-615 | 11.74 | 0 | 0 | 0 | 0 | 0 | 0 | 0 |
| cfa-miR-628 | 8230.18 | 8618.69 | 9586.13 | 10585.96 | 70796.01 | 36090.13 | 39100.42 | 40265.85 |
| cfa-miR-631 | 0 | 13.55497464 | 0 | 0 | 8.31372633 | 0 | 0 | 0 |
| cfa-miR-632 | 0 | 0 | 0 | 21.11 | 13.57 | 2.93 | 0 | 8.67 |
| cfa-miR-6516 | 2124.321882 | 2155.240968 | 2463.961628 | 1714.018673 | 1679.372719 | 1076.856294 | 1601.82079 | 941.40259 |
| cfa-miR-652 | 46117.2 | 33205.82 | 33837.62 | 33151.06 | 28072.88 | 28413.8 | 22139.6 | 22906.3 |
| cfa-miR-6529 | 281.3671367 | 189.769645 | 293.3287652 | 327.6800404 | 141.3333477 | 206.9403554 | 275.060136 | 200.973587 |
| cfa-miR-653 | 0 | 0 | 47.69 | 147.76 | 0 | 0 | 0 | 0 |
| cfa-miR-660 | 112052.6 | 104926.75 | 115868.21 | 108730.4 | 143938.78 | 140740.37 | 612.4 | 199.34 |
| cfa-miR-664 | 0 | 10.15 | 0 | 21.11 | 6.78 | 20.49 | 157173.83 | 164356.78 |
| cfa-miR-665 | 0 | 0 | 0 | 0 | 8.31372633 | 3.8322288 | 13.31 | 8.67 |
| cfa-miR-671 | 1361.91 | 1065.92 | 810.77 | 770.46 | 888.51 | 974.54 | 758.84 | 762.68 |
| cfa-miR-676 | 58.7 | 50.76 | 95.38 | 63.33 | 61.04 | 99.5 | 53.25 | 121.33 |
| cfa-miR-7 | 4858309 | 8864939.7 | 7976997.97 | 9396609.61 | 48819574.79 | 33991920.3 | 42072356.2 | 52972181 |
| cfa-miR-708 | 200377.38 | 176475.09 | 153568.9 | 148583.44 | 513308.39 | 432382.06 | 345553.44 | 445593.74 |
| cfa-miR-718 | 0 | 13.55497464 | 0 | 25.20615696 | 24.941179 | 3.8322288 | 0 | 10.5775572 |
| cfa-miR-7180 | 1913.296529 | 2643.220055 | 2522.627381 | 2760.074187 | 9760.314716 | 10293.36657 | 7523.70371 | 8007.21079 |
| cfa-miR-758 | 25711.99 | 33774.31 | 61499.1 | 78101.82 | 81648.05 | 68999.1 | 128537.47 | 141892.49 |
| cfa-miR-759 | 0 | 0 | 29.33287652 | 12.60307848 | 0 | 3.8322288 | 0 | 0 |
| cfa-miR-764 | 0 | 0 | 0 | 0 | 115.3 | 11.71 | 0 | 0 |
| cfa-miR-769 | 1153.60526 | 894.6283262 | 1671.973962 | 1487.16326 | 4472.784767 | 1854.798741 | 2087.22103 | 1480.85801 |
| cfa-miR-802 | 727.92 | 81.21 | 0 | 0 | 115.3 | 1085.75 | 812.1 | 346.67 |
| cfa-miR-872 | 0 | 0 | 0 | 0 | 0 | 0 | 0 | 10.5775572 |
| cfa-miR-874 | 53842.54 | 61366.31 | 41706.83 | 52148.8 | 260971.27 | 218961 | 141664.13 | 128840.33 |
| cfa-miR-875 | 0 | 10.15 | 0 | 0 | 0 | 0 | 0 | 0 |
| cfa-miR-876 | 70.44 | 101.52 | 143.08 | 105.54 | 718.95 | 368.74 | 359.45 | 442.01 |
| cfa-miR-8789 | 0 | 0 | 0 | 0 | 8.31372633 | 0 | 0 | 0 |
| cfa-miR-8790 | 0 | 0 | 0 | 0 | 0 | 22.99337282 | 0 | 0 |
| cfa-miR-8791a | 0 | 0 | 0 | 0 | 8.31372633 | 0 | 0 | 0 |
| cfa-miR-8791b | 0 | 13.55497464 | 29.33287652 | 12.60307848 | 0 | 0 | 16.180008 | 0 |
| cfa-miR-8794 | 56.27342733 | 0 | 0 | 0 | 8.31372633 | 0 | 0 | 0 |
| cfa-miR-8796 | 28.13671367 | 13.55497464 | 0 | 25.20615696 | 8.31372633 | 15.32891522 | 0 | 10.5775572 |
| cfa-miR-8797 | 42.2050705 | 13.55497464 | 29.33287652 | 0 | 0 | 11.49668641 | 0 | 10.5775572 |
| cfa-miR-8798 | 0 | 0 | 0 | 0 | 0 | 7.66445761 | 0 | 0 |
| cfa-miR-8799c | 0 | 0 | 0 | 0 | 0 | 0 | 0 | 10.5775572 |
| cfa-miR-8799g | 0 | 13.55497464 | 29.33287652 | 12.60307848 | 0 | 3.8322288 | 0 | 10.5775572 |
| cfa-miR-8800 | 0 | 13.55497464 | 0 | 0 | 8.31372633 | 11.49668641 | 0 | 21.1551144 |
| cfa-miR-8801 | 0 | 13.55497464 | 29.33287652 | 12.60307848 | 0 | 0 | 0 | 0 |
| cfa-miR-8802 | 14.06835683 | 13.55497464 | 29.33287652 | 12.60307848 | 0 | 3.8322288 | 0 | 10.5775572 |
| cfa-miR-8803 | 70.34178416 | 81.32984784 | 87.99862957 | 63.01539239 | 33.25490533 | 76.64457608 | 64.7200319 | 31.7326716 |
| cfa-miR-8807 | 42.2050705 | 0 | 0 | 12.60307848 | 8.31372633 | 3.8322288 | 0 | 0 |
| cfa-miR-8808 | 14.06835683 | 0 | 0 | 0 | 0 | 0 | 0 | 0 |
| cfa-miR-8809 | 0 | 13.55497464 | 29.33287652 | 37.80923544 | 8.31372633 | 0 | 16.180008 | 0 |
| cfa-miR-8810 | 0 | 13.55497464 | 0 | 0 | 0 | 0 | 0 | 0 |
| cfa-miR-8811 | 0 | 0 | 0 | 0 | 8.31372633 | 0 | 0 | 0 |
| cfa-miR-8812 | 14.06835683 | 0 | 0 | 0 | 0 | 3.8322288 | 16.180008 | 21.1551144 |
| cfa-miR-8814 | 0 | 0 | 0 | 0 | 8.31372633 | 0 | 0 | 0 |
| cfa-miR-8815 | 14.06835683 | 13.55497464 | 0 | 0 | 8.31372633 | 0 | 0 | 0 |
| cfa-miR-8816 | 0 | 0 | 0 | 0 | 0 | 3.8322288 | 0 | 10.5775572 |
| cfa-miR-8818 | 28.13671367 | 27.10994928 | 29.33287652 | 37.80923544 | 0 | 0 | 0 | 0 |
| cfa-miR-8819 | 42.2050705 | 27.10994928 | 351.9945183 | 88.22154935 | 8.31372633 | 3.8322288 | 0 | 0 |
| cfa-miR-8820 | 14.06835683 | 0 | 29.33287652 | 12.60307848 | 16.62745267 | 0 | 0 | 0 |
| cfa-miR-8822 | 14.06835683 | 0 | 0 | 0 | 0 | 0 | 0 | 0 |
| cfa-miR-8823 | 0 | 0 | 0 | 12.60307848 | 8.31372633 | 0 | 0 | 0 |
| cfa-miR-8824 | 0 | 13.55497464 | 0 | 0 | 0 | 3.8322288 | 0 | 0 |
| cfa-miR-8825 | 0 | 0 | 29.33287652 | 12.60307848 | 0 | 7.66445761 | 0 | 0 |
| cfa-miR-8826 | 0 | 13.55497464 | 58.66575305 | 37.80923544 | 615.2157487 | 674.4722695 | 760.460375 | 888.514804 |
| cfa-miR-8827 | 14.06835683 | 0 | 0 | 0 | 24.941179 | 11.49668641 | 16.180008 | 0 |
| cfa-miR-8828 | 0 | 0 | 0 | 12.60307848 | 0 | 7.66445761 | 0 | 0 |
| cfa-miR-8829 | 168.820282 | 176.2146703 | 58.66575305 | 151.2369417 | 124.705895 | 34.49005924 | 80.9000399 | 42.3102288 |
| cfa-miR-8830 | 0 | 0 | 29.33287652 | 0 | 0 | 0 | 16.180008 | 0 |
| cfa-miR-8831 | 0 | 0 | 58.66575305 | 37.80923544 | 0 | 3.8322288 | 16.180008 | 0 |
| cfa-miR-8833 | 28.13671367 | 0 | 0 | 0 | 0 | 0 | 0 | 0 |
| cfa-miR-8834a | 14.06835683 | 0 | 0 | 0 | 16.62745267 | 0 | 0 | 10.5775572 |
| cfa-miR-8834b | 14.06835683 | 0 | 29.33287652 | 0 | 0 | 0 | 0 | 0 |
| cfa-miR-8835 | 0 | 27.10994928 | 0 | 0 | 8.31372633 | 7.66445761 | 0 | 0 |
| cfa-miR-8836 | 0 | 0 | 0 | 12.60307848 | 8.31372633 | 0 | 0 | 0 |
| cfa-miR-8837 | 28.13671367 | 0 | 0 | 0 | 8.31372633 | 0 | 0 | 0 |
| cfa-miR-8838 | 14.06835683 | 0 | 0 | 12.60307848 | 8.31372633 | 0 | 0 | 0 |
| cfa-miR-8839 | 42.2050705 | 13.55497464 | 29.33287652 | 12.60307848 | 24.941179 | 22.99337282 | 0 | 0 |
| cfa-miR-8840 | 14.06835683 | 0 | 0 | 12.60307848 | 0 | 0 | 0 | 0 |
| cfa-miR-8841 | 0 | 0 | 29.33287652 | 37.80923544 | 0 | 0 | 0 | 0 |
| cfa-miR-8842 | 0 | 0 | 0 | 0 | 0 | 3.8322288 | 0 | 0 |
| cfa-miR-8843 | 0 | 13.55497464 | 29.33287652 | 25.20615696 | 0 | 0 | 0 | 0 |
| cfa-miR-8844 | 28.13671367 | 0 | 0 | 0 | 8.31372633 | 0 | 0 | 0 |
| cfa-miR-8846 | 0 | 0 | 0 | 12.60307848 | 0 | 3.8322288 | 0 | 0 |
| cfa-miR-8847 | 14.06835683 | 0 | 0 | 0 | 8.31372633 | 0 | 0 | 0 |
| cfa-miR-8848 | 0 | 0 | 0 | 0 | 0 | 3.8322288 | 0 | 0 |
| cfa-miR-8849 | 0 | 0 | 29.33287652 | 0 | 0 | 0 | 0 | 0 |
| cfa-miR-885 | 774.88 | 538.03 | 500.77 | 1160.97 | 22660.42 | 9446.88 | 7042.6 | 7895.44 |
| cfa-miR-8850 | 28.13671367 | 27.10994928 | 117.3315061 | 63.01539239 | 16.62745267 | 3.8322288 | 32.360016 | 10.5775572 |
| cfa-miR-8851 | 0 | 13.55497464 | 29.33287652 | 0 | 16.62745267 | 0 | 0 | 0 |
| cfa-miR-8853 | 14.06835683 | 0 | 0 | 0 | 0 | 3.8322288 | 0 | 0 |
| cfa-miR-8854 | 0 | 0 | 0 | 0 | 0 | 3.8322288 | 0 | 0 |
| cfa-miR-8855 | 14.06835683 | 0 | 87.99862957 | 25.20615696 | 16.62745267 | 3.8322288 | 16.180008 | 10.5775572 |
| cfa-miR-8856 | 0 | 0 | 29.33287652 | 0 | 0 | 3.8322288 | 0 | 0 |
| cfa-miR-8857 | 0 | 0 | 0 | 0 | 8.31372633 | 0 | 0 | 0 |
| cfa-miR-8858 | 0 | 0 | 0 | 0 | 8.31372633 | 3.8322288 | 0 | 0 |
| cfa-miR-8859a | 94412.7427 | 111069.4622 | 89406.60764 | 72644.14435 | 92789.49961 | 95296.03367 | 69347.5142 | 83880.0285 |
| cfa-miR-8859b | 6316.692218 | 6126.848537 | 5279.917774 | 4663.139037 | 10766.2756 | 12585.03939 | 8138.54402 | 9445.75857 |
| cfa-miR-8861 | 14.06835683 | 0 | 0 | 0 | 0 | 0 | 0 | 0 |
| cfa-miR-8863 | 0 | 0 | 0 | 0 | 8.31372633 | 0 | 0 | 10.5775572 |
| cfa-miR-8864 | 0 | 0 | 0 | 0 | 24.941179 | 0 | 0 | 0 |
| cfa-miR-8865 | 196.9569957 | 284.6544674 | 117.3315061 | 88.22154935 | 58.19608433 | 7.66445761 | 16.180008 | 52.8877859 |
| cfa-miR-8866 | 14.06835683 | 13.55497464 | 0 | 0 | 24.941179 | 3.8322288 | 0 | 0 |
| cfa-miR-8867 | 56.27342733 | 27.10994928 | 0 | 88.22154935 | 33.25490533 | 11.49668641 | 16.180008 | 0 |
| cfa-miR-8868 | 14.06835683 | 0 | 0 | 0 | 16.62745267 | 3.8322288 | 0 | 0 |
| cfa-miR-8869 | 42.2050705 | 27.10994928 | 58.66575305 | 12.60307848 | 0 | 7.66445761 | 0 | 0 |
| cfa-miR-8870 | 14.06835683 | 0 | 0 | 0 | 0 | 3.8322288 | 0 | 0 |
| cfa-miR-8871 | 14.06835683 | 0 | 0 | 0 | 0 | 0 | 0 | 10.5775572 |
| cfa-miR-8872 | 0 | 0 | 0 | 0 | 8.31372633 | 7.66445761 | 0 | 0 |
| cfa-miR-8873a | 0 | 13.55497464 | 0 | 0 | 0 | 0 | 0 | 0 |
| cfa-miR-8873b | 14.06835683 | 40.66492392 | 29.33287652 | 0 | 0 | 0 | 0 | 0 |
| cfa-miR-8874 | 28.13671367 | 13.55497464 | 58.66575305 | 0 | 8.31372633 | 0 | 0 | 0 |
| cfa-miR-8875 | 0 | 0 | 58.66575305 | 0 | 74.823537 | 3.8322288 | 16.180008 | 0 |
| cfa-miR-8876 | 42.2050705 | 94.88482248 | 0 | 88.22154935 | 133.0196213 | 80.47680489 | 64.7200319 | 52.8877859 |
| cfa-miR-8879 | 0 | 0 | 0 | 12.60307848 | 8.31372633 | 0 | 0 | 0 |
| cfa-miR-8880 | 14.06835683 | 27.10994928 | 58.66575305 | 12.60307848 | 0 | 0 | 0 | 0 |
| cfa-miR-8881 | 28.13671367 | 0 | 58.66575305 | 25.20615696 | 8.31372633 | 7.66445761 | 0 | 0 |
| cfa-miR-8882 | 28.13671367 | 0 | 0 | 25.20615696 | 0 | 0 | 0 | 0 |
| cfa-miR-8883 | 0 | 0 | 0 | 0 | 0 | 0 | 0 | 10.5775572 |
| cfa-miR-8884 | 17163.39534 | 12985.6657 | 15370.4273 | 15249.72496 | 9352.942125 | 9982.956035 | 11746.6858 | 9995.79154 |
| cfa-miR-8885 | 0 | 0 | 0 | 0 | 24.941179 | 0 | 0 | 10.5775572 |
| cfa-miR-8886 | 0 | 13.55497464 | 0 | 0 | 0 | 0 | 16.180008 | 0 |
| cfa-miR-8888 | 14.06835683 | 0 | 0 | 0 | 0 | 3.8322288 | 0 | 0 |
| cfa-miR-8889 | 14.06835683 | 13.55497464 | 0 | 0 | 0 | 0 | 0 | 0 |
| cfa-miR-889 | 231677.7003 | 285996.4099 | 502706.8378 | 677125.5974 | 613469.8662 | 509008.1264 | 1022414.7 | 1009532.64 |
| cfa-miR-8890 | 14.06835683 | 40.66492392 | 0 | 12.60307848 | 8.31372633 | 19.16114402 | 0 | 21.1551144 |
| cfa-miR-8891 | 0 | 0 | 0 | 0 | 16.62745267 | 0 | 16.180008 | 0 |
| cfa-miR-8892 | 14.06835683 | 13.55497464 | 29.33287652 | 0 | 16.62745267 | 0 | 0 | 0 |
| cfa-miR-8893 | 28.13671367 | 0 | 58.66575305 | 0 | 0 | 0 | 0 | 0 |
| cfa-miR-8894 | 42.2050705 | 0 | 0 | 0 | 8.31372633 | 7.66445761 | 16.180008 | 0 |
| cfa-miR-8895 | 0 | 0 | 0 | 12.60307848 | 0 | 0 | 0 | 0 |
| cfa-miR-8896 | 0 | 0 | 0 | 0 | 8.31372633 | 0 | 0 | 0 |
| cfa-miR-8897 | 14.06835683 | 0 | 0 | 0 | 0 | 0 | 0 | 0 |
| cfa-miR-8898 | 0 | 0 | 29.33287652 | 0 | 0 | 0 | 0 | 0 |
| cfa-miR-8899 | 14.06835683 | 0 | 0 | 0 | 0 | 0 | 0 | 0 |
| cfa-miR-8900 | 0 | 0 | 0 | 25.20615696 | 0 | 0 | 0 | 0 |
| cfa-miR-8901 | 0 | 0 | 0 | 12.60307848 | 0 | 0 | 0 | 21.1551144 |
| cfa-miR-8902 | 70.34178416 | 121.9947718 | 58.66575305 | 75.61847087 | 24.941179 | 88.14126249 | 97.0800479 | 10.5775572 |
| cfa-miR-8903 | 323.5722072 | 243.9895435 | 351.9945183 | 176.4430987 | 814.7451807 | 264.4237875 | 242.70012 | 190.396029 |
| cfa-miR-8904a | 28.13671367 | 0 | 29.33287652 | 0 | 0 | 7.66445761 | 48.540024 | 10.5775572 |
| cfa-miR-8904b | 56.27342733 | 13.55497464 | 29.33287652 | 25.20615696 | 33.25490533 | 19.16114402 | 0 | 0 |
| cfa-miR-8905 | 0 | 0 | 0 | 0 | 8.31372633 | 0 | 0 | 0 |
| cfa-miR-8907 | 0 | 0 | 0 | 0 | 0 | 7.66445761 | 0 | 0 |
| cfa-miR-8908a-3p | 28.13671367 | 13.55497464 | 29.33287652 | 0 | 2477.490447 | 2759.204739 | 4530.40224 | 1269.30686 |
| cfa-miR-8908a-5p | 0 | 0 | 29.33287652 | 0 | 515.4510327 | 655.3111255 | 1375.30068 | 349.059387 |
| cfa-miR-8908b | 14.06835683 | 0 | 0 | 12.60307848 | 565.3333907 | 620.8210663 | 1164.96058 | 264.43893 |
| cfa-miR-8908c | 0 | 0 | 0 | 0 | 0 | 26.82560163 | 0 | 21.1551144 |
| cfa-miR-8908d | 28.13671367 | 13.55497464 | 0 | 68265.21 | 798.117728 | 942.7282858 | 1650.36081 | 285.594044 |
| cfa-miR-8908e | 0 | 0 | 0 | 0 | 8.31372633 | 11.49668641 | 16.180008 | 10.5775572 |
| cfa-miR-8908f | 0 | 0 | 0 | 0 | 0 | 0 | 32.360016 | 10.5775572 |
| cfa-miR-9 | 30948.31 | 27267.15 | 24394.56 | 216384.26 | 2270769.54 | 548039.03 | 788518.44 | 812371.8 |
| cfa-miR-92a | 272464.86 | 252114.53 | 247546.38 | 35177.48 | 89698.91 | 108914.22 | 102550.4 | 97891.26 |
| cfa-miR-92b | 3510.45 | 2263.8 | 2933.07 | 72001.44 | 111667.52 | 128990.3 | 112308.86 | 84171.75 |
| cfa-miR-93 | 248431.79 | 221700.39 | 226800.27 | 153427.86 | 96047.36 | 95806.23 | 90581.97 | 89380.48 |
| cfa-miR-95 | 30549.13 | 33276.88 | 29306.86 | 23698720.77 | 47877.85 | 31697.38 | 23657.28 | 13823.51 |
| cfa-miR-96 | 150808.43 | 157603.3 | 99676.71 | 2286430.83 | 1437373.37 | 2083438 | 2440241.53 | 1787999.7 |
| cfa-miR-98 | 157488.85 | 144984.89 | 136757.39 | 153427.86 | 242610.97 | 175820.84 | 178900.72 | 185503.72 |
| cfa-miR-99a | 19810873.45 | 23061916.43 | 23885090.18 | 23698720.77 | 19940044.75 | 27288015.58 | 25061757.2 | 21580416.7 |
| cfa-miR-99b | 2002270.97 | 2051421.46 | 2195129.06 | 2286430.83 | 1092230.94 | 1491354.5 | 1430473.56 | 1199846.12 |
