# Supplementary material for: Comparison of miRNA expression profiles in pituitary–adrenal axis between Beagle and Chinese Field dogs after chronic stress exposure
Source: PeerJ. 2016 Feb 18;4:e1682. doi: 10.7717/peerj.1682 (PMC4768678; doi:10.7717/peerj.1682)
Supplement: Table S4 [file peerj-04-1682-s007.docx]

**Table S4.** Top 20 miRNAs expressing with the highest abundance in four adrenal cortex samples and four pituitary samples, respectively.

| **MiRNAs** | **CFDAC1** | **CFDAC2** | **BAC1** | **BAC2** | **Total normalized reads in four libraries** |
| --- | --- | --- | --- | --- | --- |
| cfa-miR-99a | 19810873 | 23061916 | 23885090 | 23698721 | 90456601 |
| cfa-miR-21 | 11362479 | 8917961 | 8270830 | 9166959 | 37718228 |
| cfa-miR-10b | 8593051 | 10484330 | 10830756 | 7734339 | 37642477 |
| cfa-miR-26a | 9605728 | 9394091 | 9373879 | 8741314 | 37115012 |
| cfa-miR-143 | 10111291 | 7168042 | 9142691 | 9274211 | 35696235 |
| cfa-miR-7 | 4858309 | 8864940 | 7976998 | 9396610 | 31096856 |
| cfa-miR-374a | 8402231 | 7377764 | 6606706 | 8459683 | 30846383 |
| cfa-miR-202 | 7718715 | 7747423 | 7427822 | 6877351 | 29771311 |
| cfa-miR-30a | 5489756 | 4844690 | 5658608 | 4963782 | 20956836 |
| cfa-miR-27b | 5225157 | 4532651 | 5122167 | 5830649 | 20710623 |
| cfa-miR-30d | 5056338 | 5151400 | 4390377 | 3785722 | 18383837 |
| cfa-miR-29a | 3936458 | 3129053 | 3368500 | 3569169 | 14003180 |
| cfa-miR-186 | 3402541 | 3581630 | 2984769 | 2481664 | 12450604 |
| cfa-miR-125b | 2674399 | 3404810 | 3009473 | 3206618 | 12295300 |
| cfa-miR-145 | 3740812 | 2661095 | 2467809 | 2956808 | 11826525 |
| cfa-let-7g | 3306573 | 2869081 | 2758636 | 2813196 | 11747486 |
| cfa-miR-26b | 2753613 | 2877933 | 2535795 | 2246272 | 10413612 |
| cfa-miR-22 | 2820006 | 2040488 | 2290490 | 2258853 | 9409836 |
| cfa-miR-30b | 2618783 | 2307718 | 2432732 | 1863288 | 9222521 |
| cfa-miR-30c | 2397461 | 2274980 | 2359143 | 2076643 | 9108226 |
| **MiRNAs** | **CFDP1** | **CFDP2** | **BP1** | **BP2** | **Total normalized reads in four pituitary libraries** |
| cfa-miR-7 | 48819575 | 33991920 | 42072356 | 52972181 | 1.78E+08 |
| cfa-miR-99a | 19940045 | 27288016 | 25061757 | 21580417 | 93870234 |
| cfa-miR-375 | 10726742 | 12287974 | 9585913 | 11114511 | 43715139 |
| cfa-miR-26a | 7900619 | 7717516 | 6396173 | 6323871 | 28338179 |
| cfa-miR-125b | 5966317 | 7841985 | 4403503 | 4879717 | 23091522 |
| cfa-miR-374a | 2786703 | 4550098 | 7711742 | 3956627 | 19005170 |
| cfa-miR-30a | 2553208 | 5289957 | 6285675 | 4436273 | 18565112 |
| cfa-miR-335 | 4575893 | 4631149 | 4001569 | 4075891 | 17284501 |
| cfa-miR-29a | 4012136 | 3820760 | 4014629 | 4175758 | 16023282 |
| cfa-miR-27b | 3322590 | 4124008 | 3439612 | 3324949 | 14211159 |
| cfa-miR-141 | 2315039 | 3512334 | 4626710 | 3372330 | 13826413 |
| cfa-miR-30d | 3332493 | 3599250 | 2632735 | 3220913 | 12785390 |
| cfa-miR-148a | 2048045 | 1881416 | 2807003 | 3022652 | 9759115 |
| cfa-miR-96 | 1437373 | 2083438 | 2440242 | 1788000 | 7749053 |
| cfa-let-7g | 1938222 | 1773453 | 1902394 | 1849690 | 7463759 |
| cfa-miR-411 | 1849683 | 1745118 | 1911780 | 1837132 | 7343713 |
| cfa-miR-24 | 1868057 | 2044620 | 1717836 | 1667696 | 7298209 |
| cfa-miR-181a | 2144384 | 2085171 | 1600921 | 1297382 | 7127857 |
| cfa-miR-21 | 2339782 | 1411952 | 1716411 | 1605079 | 7073223 |
| cfa-miR-125a | 1729022 | 1935352 | 1108391 | 1323946 | 6096711 |

BAC1, Beagle adrenal cortex with treatment; BAC2, Beagle adrenal cortex without treatment; BP1, Beagle pituitary with treatment; BP2, Beagle pituitary without treatment; CFDAC1, Chinese Field Dog adrenal cortex with treatment; CFDAC2, Chinese Field Dog adrenal cortex without treatment; CFDP1, Chinese Field Dog pituitary with treatment; CFDP2, Chinese Field Dog pituitary without treatment.
