# Supplementary material for: Comparison of miRNA expression profiles in pituitary–adrenal axis between Beagle and Chinese Field dogs after chronic stress exposure
Source: PeerJ. 2016 Feb 18;4:e1682. doi: 10.7717/peerj.1682 (PMC4768678; doi:10.7717/peerj.1682)
Supplement: Table S5 [file peerj-04-1682-s008.docx]

Table S5. Novel miRNAs predicted by miRDeep2.

| **Provisional id** | **Mature read count** | **Example miRBase miRNA with the same seed** | **Consensus mature sequence** | **Precursor coordinate** |
| --- | --- | --- | --- | --- |
| chr24_20231 | 102626 | aca-miR-1388-5p | aggacuguccaaccugagagu | chr24:47411218..47411275:- |
| chr21_17074 | 22949 | ppy-miR-1226 | ccaccagcuggcguucccugg | chr21:20592019..20592078:+ |
| chr28_23636 | 14328 | hsa-miR-593-5p | uggcaccagcacuggcggugg | chr28:27372109..27372165:- |
| chrX_41704 | 11332 | hsa-miR-4530 | accagcagaggcuuggagcagg | chrX:32945419..32945483:- |
| chr38_30769 | 3043 | rno-miR-215 | augaccuacgaauugauagaca | chr38:14895116..14895175:- |
| chrX_41456 | 2639 | hsa-miR-2114-5p | uagucccuuccuugaaggaucgg | chrX:118541359..118541417:+ |
| chr8_37788 | 1170 | gma-miR4381 | uaugugacaugguccacuaau | chr8:69256925..69256981:+ |
| chr31_26916 | 1103 | mmu-miR-670-3p | uuuccucacagugugggucugu | chr31:32635980..32636042:+ |
| chr10_2999 | 839 | ahy-miR3511-5p | cccagggccgaccgcgcaggucg | chr10:16859590..16859655:- |
| chr6_35394 | 668 | sbi-miR6222-5p | ucuguuugcucucaccuucagg | chr6:38960634..38960716:- |
| chr24_19855 | 666 | oan-miR-1388-3p | cucucagguuggacaguccugga | chr24:47411220..47411279:+ |
| chr31_26701 | 568 | hvt-miR-H12-5p | uuucuuauuucuuccaccgaga | chr31:10386528..10386590:+ |
| chr31_27046 | 568 | hvt-miR-H12-5p | uuucuuauuucuuccaccgaga | chr31:10386525..10386587:- |
| chr24_20048 | 392 | dme-miR-14-3p | ucagucuuuggccugccucucc | chr24:22892648..22892709:- |
| chr2_14652 | 313 | bta-miR-2284i | gagaaguuucucugaacgugu | chr2:6117328..6117383:- |
| chr20_16009 | 300 | rno-miR-6318 | cugccuggaccccugcucacc | chr20:50760565..50760623:+ |
| chr15_9122 | 232 | ebv-miR-BART7-5p | gcuggaccuugaauauccuauau | chr15:23529218..23529285:- |
| chr19_13764 | 200 | mmu-miR-361-3p | ucccccagcagugguaagcc | chr19:53244978..53245034:- |
| chr16_9309 | 193 | gga-miR-6547-3p | uccccuccucgccuucccucagg | chr16:94389..94471:+ |
| chr2_14055 | 187 | ptr-miR-4742 | ucaggcaagacgauguggcaagu | chr2:33930541..33930602:+ |
| chr11_4085 | 183 | ppy-miR-136 | ucuccauugaacgaugcucccc | chr11:63290160..63290221:+ |
| chr27_22971 | 162 | aca-miR-5468 | auuggaguuacuuggauaaaga | chr27:40981548..40981601:- |
| chr22_18230 | 157 | osa-miR397b | uuauugaguuuuguauuuuucu | chr22:3273074..3273129:- |
| chr12_4913 | 133 | sme-miR-184-5p | ucuccuuugcaucacuaccacag | chr12:1123075..1123142:+ |
| chr11_4686 | 122 | aly-miR869-3p | ccuggguucugucuccacucugc | chr11:55410564..55410623:- |
| chr15_9068 | 119 | oan-miR-449b-3p | aagccaccccgagcagcagcgu | chr15:19124914..19124961:- |
| chr3_24636 | 117 | gga-miR-1746 | uucagagcccuccuacaauguga | chr3:35437742..35437807:+ |
| chr32_27732 | 109 | cin-miR-4024-3p | uuucaucuaucuuaucaaacu | chr32:22278068..22278125:- |
| chr30_26337 | 106 | xtr-miR-124 | uuaaggcaccugagucagaccgg | chr30:10421046..10421110:- |
| chr10_2411 | 104 | cel-miR-2218a-3p | uggccagacggaggacugagu | chr10:21445010..21445066:+ |
| chr27_22512 | 102 | gga-miR-6596-5p | uccgagcccgggucucccucu | chr27:1230452..1230515:- |
| chr10_2522 | 89 | bta-miR-1835 | ugcacccugagagcuggagcag | chr10:27296054..27296115:+ |
| chr2_14511 | 83 | mmu-miR-18a-3p | ucugcccuccguguguccccaga | chr2:77542007..77542071:+ |
| chr38_30571 | 80 | mtr-miR5208b | cacauggaguugcuguuacacu | chr38:14895403..14895461:+ |
| chr3_24658 | 80 | ggo-miR-2110 | auggggaaccguuuuuguucuu | chr3:39849991..39850054:+ |
| chr10_3064 | 78 | cel-miR-789 | ucccugcccucugcccaccagg | chr10:23893782..23893847:- |
| chr2_15105 | 75 | hsa-miR-4274 | uagcagucucugcagaggaaaga | chr2:65930314..65930375:- |
| chr19_13409 | 70 | ppy-miR-1915 | ccccagggacccgggaaccagc | chr19:38303252..38303319:+ |
| chr38_30570 | 69 | aly-miR402-5p | uuggccuacaaaaaugacagac | chr38:14895118..14895178:+ |
| chr1_829 | 54 | hsa-miR-371b-3p | aagugcccccacaguuugagugg | chr1:103606523..103606583:+ |
| chr1_823 | 54 | hsa-miR-371b-3p | aagugcccccacaguuugagugg | chr1:103406620..103406680:+ |
| chr3_25089 | 52 | bta-miR-2434 | uuguuggguguccuccugcagc | chr3:91623824..91623882:+ |
| chr13_6545 | 50 | hsa-miR-5008-3p | gcugugcucuucucuccccaga | chr13:37816081..37816136:+ |
| chrX_42258 | 50 | gga-miR-1802 | uagugacugcaagccugaguaga | chrX:114717102..114717160:- |
| chr9_39419 | 48 | hsa-miR-5581-5p | ugccuuccccugugcuguagg | chr9:48626063..48626117:+ |
| chr4_30923 | 43 | gga-miR-1617 | ugaggccucuggcgggcagccaa | chr4:5155127..5155188:+ |
| chr5_32351 | 43 | dps-miR-2535-3p | gcucacgggaguucuugguuuc | chr5:15196614..15196677:+ |
| chr5_33705 | 39 | cin-miR-4006a-3-3p | aucauuaccaggcaguauuaga | chr5:56368406..56368463:- |
| chr2_14385 | 36 | osa-miR167d-5p | agaagcugugguuggagccggc | chr2:69438625..69438686:+ |
| chr10_2506 | 35 | hsa-miR-4437 | ugggcucagacuuuagcugugu | chr10:26687388..26687450:+ |
| chr17_11368 | 34 | aly-miR156f-3p | ucucacucuagucccagaau | chr17:21618746..21618804:- |
| chr28_23508 | 31 | hsa-miR-629-3p | cuucucccugcugucucucagg | chr28:14227439..14227498:- |
| chr6_35448 | 31 | cgr-miR-484 | ucaggcucucggaaggucaugc | chr6:40256202..40256261:- |
| chr6_35138 | 29 | rlcv-miR-rL1-29-5p | ucagucccccuacugccugcagc | chr6:17239256..17239319:- |
| chr25_20958 | 28 | rno-miR-347 | ugucccucagaccuaccugga | chr25:30915421..30915481:- |
| chr18_12331 | 28 | mmu-miR-344h-5p | uaggcuuccugacucugugggacu | chr18:52084972..52085031:+ |
| chr7_35747 | 28 | cre-miR1161a | gacuggaggacuuagguugcacg | chr7:449942..450015:+ |
| chr15_8515 | 28 | aly-miR4248b | ucauuuuaaagagauuccauca | chr15:30155122..30155182:+ |
| chr2_14133 | 25 | aca-miR-5402-3p | uucuguagucaucuuugauaga | chr2:37378351..37378418:+ |
| chr2_14065 | 24 | gga-miR-1649-3p | guccgugcacgucuccaaccugc | chr2:34434769..34434831:+ |
| chr24_20233 | 22 | dme-miR-1015-3p | uccugggacuuguaguucgcgg | chr24:47423544..47423618:- |
| chr1_621 | 19 | csi-miR857 | uuuugaaucagccaucccuuugu | chr1:83242684..83242743:+ |
| chr20_16272 | 19 | aca-miR-5457 | ccaacagcaugaccggcagugu | chr20:3513378..3513439:- |
| chr9_38831 | 17 | hsa-miR-2964a-3p | ugaauugcuggccucuccccagcu | chr9:6816748..6816819:+ |
| chr6_35710 | 16 | nta-miR6145b | auaucauaugauucaacguagu | chr6:71962802..71962879:- |
| chr10_2996 | 13 | mmu-miR-337-3p | aucagcuccugaaacuccccucaga | chr10:16647734..16647799:- |
| chr20_15410 | 12 | ppt-miR902d-3p | cugaagguaggaugugaugaga | chr20:3821730..3821788:+ |
| chr18_12339 | 11 | hsa-miR-5190 | ucagugacaauuccuggcaga | chr18:52326739..52326803:+ |
| chr22_18506 | 9 | hsa-miR-4762-3p | uuucugauaagccccuugucc | chr22:56305775..56305838:- |
| chr12_6096 | 8 | mml-miR-544 | auucugcauuuguaacaagcuc | chr12:49638410..49638468:- |
| chr34_28509 | 8 | hsa-miR-5698 | ggggggagagggaggagga | chr34:17078144..17078201:+ |
| chr13_7041 | 7 | gga-miR-1615 | gggcagcugcuuuguccugugugc | chr13:36492902..36492966:- |
| chr5_32241 | 5 | osa-miR5833 | accuccucucuuugcaaacagg | chr5:4980809..4980872:+ |
| chrX_41033 | 4 | ggo-miR-4488 | cagggggcccccagcccgggaucc | chrX:55470425..55470488:+ |
| chr8_37811 | 20860 | - | aaaaauacgggugcacuucugu | chr8:69266325..69266383:+ |
| chrX_41019 | 9478 | - | aacagccucuggcauguugg | chrX:53926202..53926246:+ |
| chrX_42318 | 4697 | - | uccacggugguggaauugucc | chrX:119928559..119928619:- |
| chrX_41069 | 2769 | - | uuacaauacaaccugguaagu | chrX:57590396..57590444:+ |
| chrX_42248 | 1643 | - | aauuaggaccucccugagcgga | chrX:114684099..114684159:- |
| chrX_42244 | 1643 | - | aauuaggaccucccugagcgga | chrX:114680910..114680970:- |
| chrX_42256 | 1643 | - | aauuaggaccucccugagcgga | chrX:114689812..114689872:- |
| chrX_42264 | 1934 | - | ugauuggcaccucuuugagugu | chrX:115652721..115652779:- |
| chrX_42252 | 1643 | - | aauuaggaccucccugagcgga | chrX:114687029..114687089:- |
| chrX_42262 | 1177 | - | ugaauggcaccuuuuugaguagg | chrX:115652424..115652482:- |
| chr18_12467 | 891 | - | uaucuguggaauacucauucuca | chr18:4871031..4871094:- |
| chr28_23510 | 761 | - | uuuuggcuggaccugccccagc | chr28:14231916..14231975:- |
| chrX_42242 | 666 | - | uuagcgccugacugaguggggu | chrX:114679974..114680034:- |
| chrX_42254 | 666 | - | uuagcgccugacugaguggggu | chrX:114688875..114688935:- |
| chrX_42250 | 666 | - | uuagcgccugacugaguggggu | chrX:114686167..114686227:- |
| chrX_42240 | 385 | - | gauuaguuccugccuggauaca | chrX:114678504..114678561:- |
| chrUn_JH373668_42827 | 385 | - | gauuaguuccugccuggauaca | chrUn_JH373668:7415..7472:+ |
| chrX_42266 | 503 | - | acugucaccuuuuuugaguac | chrX:115658398..115658456:- |
| chrX_42268 | 503 | - | acugucaccuuuuuugaguac | chrX:115658609..115658666:- |
| chrUn_JH373668_42836 | 320 | - | uauccagaaaggcgcucguuau | chrUn_JH373668:13007..13064:+ |
| chr24_19732 | 477 | - | uucuccuccucccagccaggu | chr24:39048206..39048264:+ |
| chr6_35078 | 337 | - | uggcagcaugaugccagggcagc | chr6:12879001..12879058:- |
| chr37_30159 | 141 | - | gguuagggcgcgucacgugacg | chr37:25734791..25734852:+ |
| chr25_20311 | 290 | - | ugaaaaagccacguguaagugg | chr25:7638707..7638764:+ |
| chr9_39445 | 260 | - | ucccaaggccugacacagcucc | chr9:49018359..49018417:+ |
| chr15_8855 | 250 | - | guccucuugaguccugauggguu | chr15:3645321..3645378:- |
| chr9_39220 | 150 | - | aggacuacggacgggcugagcu | chr9:32760904..32760964:+ |
| chr10_2324 | 242 | - | uuaaacuugauuuagcuuuuccu | chr10:13303676..13303732:+ |
| chr3_25502 | 225 | - | uuuccacccuggcccugcagg | chr3:53296008..53296070:- |
| chr3_24487 | 160 | - | ccugagagugaguaccuccuga | chr3:15387060..15387123:+ |
| chr30_26451 | 213 | - | aagcuacccggaugccccu | chr30:23619737..23619803:- |
| chrUn_JH373668_42833 | 210 | - | gauuagcaucugccugggcaga | chrUn_JH373668:10366..10423:+ |
| chr10_2708 | 192 | - | ccauccuaaggugucugugga | chr10:48900710..48900770:+ |
| chr9_38869 | 182 | - | aaaauuaccugugucccuugau | chr9:9465144..9465201:+ |
| chr31_26762 | 175 | - | uucucauuggcuucauguccugu | chr31:24034698..24034749:+ |
| chr33_28379 | 174 | - | aggcauuagcagauccgaacu | chr33:29705322..29705391:- |
| chr22_17793 | 157 | - | uuauugaguuuuguauuuuucu | chr22:3273077..3273132:+ |
| chr14_7814 | 123 | - | ucugagcugccuuuuccuuuu | chr14:3837162..3837224:- |
| chr23_18767 | 139 | - | auguucaaauuguuuuguagg | chr23:24660156..24660211:+ |
| chr20_15741 | 99 | - | ccgccacaauucccucuggc | chr20:40145530..40145587:+ |
| chr30_26128 | 118 | - | acagcggaggaaacagagauccu | chr30:29119213..29119279:+ |
| chr11_4176 | 106 | - | auccuaagguuggacggucugg | chr11:74256390..74256446:+ |
| chr7_36519 | 105 | - | uuaaguaaacuaugauucauc | chr7:571634..571693:- |
| chr4_31326 | 102 | - | cauccauuucuuucaccugggg | chr4:50474028..50474086:+ |
| chr4_31140 | 93 | - | ugauccucuggacaccuccaga | chr4:26004697..26004761:+ |
| chr31_26945 | 62 | - | cccucccggguaccuguuccagg | chr31:36627354..36627416:+ |
| chr9_40075 | 93 | - | ugggguagugagaggccaggucu | chr9:23524552..23524608:- |
| chr23_18861 | 77 | - | gaauguggcagucucucugagc | chr23:36551679..36551733:+ |
| chr20_15729 | 77 | - | ucaggcuuauucuggcccuggcagg | chr20:40081098..40081160:+ |
| chr23_19246 | 76 | - | gaauguggcagucucucugagc | chr23:36551677..36551731:- |
| chr31_26870 | 64 | - | aacucgaugcaauucacuuugug | chr31:29080845..29080899:+ |
| chr2_15135 | 40 | - | acuccccccuugcucaccucuccu | chr2:68975011..68975075:- |
| chr31_26824 | 64 | - | aacucgaugcaauucacuuugug | chr31:28181996..28182050:+ |
| chr20_16902 | 31 | - | cggccgcggggucuccugcccgc | chr20:57809622..57809684:- |
| chr25_21015 | 37 | - | ugaacuacccgugagaggucau | chr25:38921454..38921514:- |
| chr25_20576 | 37 | - | ugaacuacccgugagaggucau | chr25:38921457..38921517:+ |
| chr34_28835 | 46 | - | cucucucuccccuccuuuucucu | chr34:32454260..32454324:- |
| chr23_18931 | 35 | - | uaggaguuaacaugugaguuga | chr23:46798849..46798907:+ |
| chr38_30767 | 38 | - | aaacaguugguaauuuucagac | chr38:14874844..14874906:- |
| chr20_15393 | 40 | - | ugacccuccgcccugagacaccc | chr20:2801008..2801069:+ |
| chr6_35433 | 30 | - | cccccaccccaggucuagcucu | chr6:39910577..39910636:- |
| chr9_38755 | 36 | - | uaccuggugcgccuaggacgagg | chr9:2248477..2248537:+ |
| chr8_37543 | 35 | - | uaccauacagggauuccuuuua | chr8:46970530..46970577:+ |
| chr28_23724 | 20 | - | ucccgggcgccugcugaggau | chr28:40038036..40038091:- |
| chr20_16098 | 25 | - | uaaguccugcaggaggagaagc | chr20:54655272..54655333:+ |
| chr23_18823 | 22 | - | ugcccucugugauucacgcaga | chr23:31862574..31862638:+ |
| chr3_25462 | 24 | - | uggccccucugccggaggucuga | chr3:48131548..48131607:- |
| chr9_40413 | 24 | - | caacuccagggggugcuguuc | chr9:46179588..46179644:- |
| chr7_36330 | 25 | - | ugugcuucccgagcuccucauu | chr7:63206311..63206370:+ |
| chr26_21538 | 20 | - | guccuucucccguccgucugcu | chr26:27769628..27769692:+ |
| chr3_24700 | 18 | - | ugccggguuccucagggcac | chr3:46345869..46345930:+ |
| chr10_2347 | 13 | - | ugcuccccucugcuccugcagg | chr10:16831460..16831534:+ |
| chr24_19615 | 18 | - | acgcugaccccaccucucag | chr24:27295901..27295949:+ |
