# Supplementary material for: Comparison of miRNA expression profiles in pituitary–adrenal axis between Beagle and Chinese Field dogs after chronic stress exposure
Source: PeerJ. 2016 Feb 18;4:e1682. doi: 10.7717/peerj.1682 (PMC4768678; doi:10.7717/peerj.1682)
Supplement: Table S6 [file peerj-04-1682-s009.docx]

Table S6. Novel miRNAs predicted by miRDeep2, with the expression levels greater than 1000 reads.

| **Provisional id** | **Mature read count** | **Example miRBase miRNA with the same seed** | **Consensus mature sequence** | **Precursor coordinate** |
| --- | --- | --- | --- | --- |
| **chr24_20231** | 102626 | aca-miR-1388-5p | aggacuguccaaccugagagu | chr24:47411218..47411275:- |
| **chr21_17074** | 22949 | ppy-miR-1226 | ccaccagcuggcguucccugg | chr21:20592019..20592078:+ |
| **chr8_37811** | 20860 | - | aaaaauacgggugcacuucugu | chr8:69266325..69266383:+ |
| **chr28_23636** | 14328 | hsa-miR-593-5p | uggcaccagcacuggcggugg | chr28:27372109..27372165:- |
| **chrX_41704** | 11332 | hsa-miR-4530 | accagcagaggcuuggagcagg | chrX:32945419..32945483:- |
| **chrX_41019** | 9478 | - | aacagccucuggcauguugg | chrX:53926202..53926246:+ |
| **chrX_42318** | 4697 | - | uccacggugguggaauugucc | chrX:119928559..119928619:- |
| **chr38_30769** | 3043 | rno-miR-215 | augaccuacgaauugauagaca | chr38:14895116..14895175:- |
| **chrX_41069** | 2769 | - | uuacaauacaaccugguaagu | chrX:57590396..57590444:+ |
| **chrX_41456** | 2639 | hsa-miR-2114-5p | uagucccuuccuugaaggaucgg | chrX:118541359..118541417:+ |
| **chrX_42264** | 1934 | - | ugauuggcaccucuuugagugu | chrX:115652721..115652779:- |
| **chrX_42248** | 1643 | - | aauuaggaccucccugagcgga | chrX:114684099..114684159:- |
| **chrX_42244** | 1643 | - | aauuaggaccucccugagcgga | chrX:114680910..114680970:- |
| **chrX_42256** | 1643 | - | aauuaggaccucccugagcgga | chrX:114689812..114689872:- |
| **chrX_42252** | 1643 | - | aauuaggaccucccugagcgga | chrX:114687029..114687089:- |
| **chrX_42262** | 1177 | - | ugaauggcaccuuuuugaguagg | chrX:115652424..115652482:- |
| **chr8_37788** | 1170 | gma-miR4381 | uaugugacaugguccacuaau | chr8:69256925..69256981:+ |
| **chr31_26916** | 1103 | mmu-miR-670-3p | uuuccucacagugugggucugu | chr31:32635980..32636042:+ |
