# Supplementary material for: Comparison of miRNA expression profiles in pituitary–adrenal axis between Beagle and Chinese Field dogs after chronic stress exposure
Source: PeerJ. 2016 Feb 18;4:e1682. doi: 10.7717/peerj.1682 (PMC4768678; doi:10.7717/peerj.1682)
Supplement: Table S7 [file peerj-04-1682-s010.docx]

Table S7. Differentially expressed miRNA of BAC1_vs_BAC2, BAC1_vs_CFDAC1, BAC2_vs_CFDAC2, CFDAC1_vs_CFDAC2.

| **BAC1_vs_BAC2; 27 (6 up, 21 down) DE-miRNAs; P-value < 0.001, log2(Fold_change) >1** | | | | | | | | |
| --- | --- | --- | --- | --- | --- | --- | --- | --- |
| **MiRNAs** | | **BAC1** | **BAC2** | **log2(Fold_change)** | **z-score** | **p-value** | **Signature(p-value < 0.001)** | |
| cfa-miR-338 | | 62452.94 | 168721.04 | -1.433798499 | -225.1699 | 0 | TRUE | |
| cfa-miR-9 | | 24394.56 | 68265.21 | -1.484591063 | -146.9764 | 0 | TRUE | |
| cfa-miR-138a | | 10468.44 | 35789.63 | -1.77349516 | -120.9194 | 0 | TRUE | |
| cfa-miR-124 | | 6199.99 | 24559.86 | -1.985964543 | -108.1139 | 0 | TRUE | |
| cfa-miR-122 | | 13067.66 | 591.04 | 4.466601242 | 115.41862 | 0 | TRUE | |
| cfa-miR-196b | | 10229.98 | 1931.44 | 2.405054558 | 78.602744 | 0 | TRUE | |
| cfa-miR-138b | | 1216.15 | 4242.83 | -1.802705693 | -42.10384 | 0 | TRUE | |
| cfa-miR-490 | | 1096.92 | 2406.38 | -1.13340617 | -22.38516 | 5.49E-111 | TRUE | |
| cfa-miR-31 | | 786.92 | 2290.28 | -1.541235106 | -27.67233 | 1.50E-168 | TRUE | |
| cfa-miR-496 | | 977.69 | 2068.64 | -1.081233597 | -19.97905 | 8.38E-89 | TRUE | |
| cfa-miR-206 | | 2217.69 | 707.14 | 1.648989938 | 28.606573 | 5.57E-180 | TRUE | |
| cfa-miR-885 | | 500.77 | 1160.97 | -1.213110651 | -16.41391 | 1.52E-60 | TRUE | |
| cfa-miR-493 | | 405.38 | 833.79 | -1.040409156 | -12.29169 | 1.00E-34 | TRUE | |
| cfa-miR-196a | | 858.46 | 84.43 | 3.345923294 | 26.910546 | 1.65E-159 | TRUE | |
| cfa-miR-135a-3p | | 286.15 | 654.37 | -1.193335002 | -12.16357 | 4.86E-34 | TRUE | |
| cfa-miR-216b | | 214.61 | 517.16 | -1.268893392 | -11.34868 | 7.53E-30 | TRUE | |
| cfa-miR-33b | | 214.61 | 443.28 | -1.04650097 | -9.005332 | 2.15E-19 | TRUE | |
| cfa-miR-653 | | 47.69 | 147.76 | -1.631497084 | -7.325002 | 2.39E-13 | TRUE | |
| cfa-miR-205 | | 47.69 | 116.1 | -1.283609284 | -5.425628 | 5.78E-08 | TRUE | |
| cfa-miR-551b | | 119.23 | 31.66 | 1.913014124 | 7.3506451 | 1.97E-13 | TRUE | |
| cfa-miR-371 | | 23.85 | 94.99 | -1.993786377 | -6.740794 | 1.58E-11 | TRUE | |
| cfa-miR-1839 | | 23.85 | 63.33 | -1.408899813 | -4.303943 | 1.68E-05 | TRUE | |
| cfa-miR-1840 | | 47.69 | 0 | 6.575614878 | 7.1222514 | 1.06E-12 | TRUE | |
| cfa-miR-514 | | 0 | 31.66 | -5.98458935 | -5.850485 | 4.90E-09 | TRUE | |
| cfa-miR-448 | | 0 | 21.11 | -5.399854674 | -4.770143 | 1.84E-06 | TRUE | |
| cfa-miR-632 | | 0 | 21.11 | -5.399854674 | -4.770143 | 1.84E-06 | TRUE | |
| cfa-miR-664 | | 0 | 21.11 | -5.399854674 | -4.770143 | 1.84E-06 | TRUE | |
|  | |  |  |  |  |  |  | |
| **BAC1_vs_CFDAC1; 37 (18 up, 19 down) DE-miRNAs; P-value < 0.001, log2(Fold_change) >1** | | | | | | | | |
| **MiRNAs** | | **BAC1** | **CFDAC1** | **log2(Fold_change)** | **z-score** | **p-value** | **Signature(p-value < 0.001)** | |
| cfa-miR-451 | | 1143897.5 | 2376996.7 | -1.055182141 | -667.6324 | 0 | TRUE | |
| cfa-miR-411 | | 873005.79 | 413505.08 | 1.078086169 | 410.07717 | 0 | TRUE | |
| cfa-miR-889 | | 502706.84 | 231677.7 | 1.117598126 | 72.857056 | 0 | TRUE | |
| cfa-miR-34a | | 84319.82 | 197301.33 | -1.226456989 | -216.0654 | 0 | TRUE | |
| cfa-miR-379 | | 136590.47 | 64456.07 | 1.083468697 | 162.62689 | 0 | TRUE | |
| cfa-miR-222 | | 37128.38 | 94946.5 | -1.354592451 | -161.8704 | 0 | TRUE | |
| cfa-miR-758 | | 61499.1 | 25711.99 | 1.258124025 | 122.92577 | 0 | TRUE | |
| cfa-miR-221 | | 22558.41 | 50942.6 | -1.175207209 | -106.1133 | 0 | TRUE | |
| cfa-miR-223 | | 4745.37 | 10543.09 | -1.151705274 | -47.49846 | 0 | TRUE | |
| cfa-miR-196b | | 10229.98 | 2254.2 | 2.182115898 | 74.096987 | 0 | TRUE | |
| cfa-miR-592 | | 4316.14 | 2031.13 | 1.087459081 | 28.987688 | 9.41E-185 | TRUE | |
| cfa-miR-31 | | 786.92 | 1890.24 | -1.264280541 | -21.65001 | 6.08E-104 | TRUE | |
| cfa-miR-138b | | 1216.15 | 598.77 | 1.022247336 | 14.628283 | 1.85E-48 | TRUE | |
| cfa-miR-490 | | 1096.92 | 493.11 | 1.153476896 | 15.32466 | 5.23E-53 | TRUE | |
| cfa-miR-217 | | 333.85 | 962.73 | -1.527931207 | -17.83761 | 3.61E-71 | TRUE | |
| cfa-miR-196a | | 858.46 | 399.18 | 1.104711474 | 13.093746 | 3.58E-39 | TRUE | |
| cfa-miR-105b | | 357.69 | 798.36 | -1.158329654 | -13.1304 | 2.20E-39 | TRUE | |
| cfa-miR-507a | | 263.99589 | 886.30648 | -1.747290198 | -3.610297 | 0.0001529 | TRUE | |
| cfa-miR-216b | | 214.61 | 751.4 | -1.807863814 | -17.76194 | 1.39E-70 | TRUE | |
| cfa-miR-34c | | 667.69 | 270.03 | 1.306058735 | 13.18517 | 1.07E-39 | TRUE | |
| cfa-miR-802 | | 190.77 | 727.92 | -1.931945589 | -18.28433 | 1.10E-74 | TRUE | |
| cfa-miR-33b | | 214.61 | 598.77 | -1.480284639 | -13.74189 | 5.70E-43 | TRUE | |
| cfa-miR-493 | | 405.38 | 187.85 | 1.109693797 | 9.0305661 | 1.71E-19 | TRUE | |
| cfa-miR-133b | | 143.08 | 293.52 | -1.036636786 | -7.277364 | 3.40E-13 | TRUE | |
| cfa-miR-8819 | | 351.99452 | 42.205071 | 3.060064722 | 3.9375147 | 8.23E-05 | TRUE | |
| cfa-miR-383 | | 286.15 | 93.93 | 1.607113693 | 10.082232 | 6.62E-24 | TRUE | |
| cfa-miR-208b | | 286.15 | 82.18 | 1.799912371 | 10.92114 | 9.13E-28 | TRUE | |
| cfa-miR-876 | | 143.08 | 70.44 | 1.022355209 | 5.0179465 | 5.22E-07 | TRUE | |
| cfa-miR-489 | | 119.23 | 46.96 | 1.344242971 | 5.6968831 | 1.22E-08 | TRUE | |
| cfa-miR-551b | | 119.23 | 35.22 | 1.75928047 | 6.9390857 | 3.95E-12 | TRUE | |
| cfa-miR-200b | | 23.85 | 129.15 | -2.436986472 | -8.900375 | 5.57E-19 | TRUE | |
| cfa-miR-1839 | | 23.85 | 93.93 | -1.977596743 | -6.670269 | 2.55E-11 | TRUE | |
| cfa-miR-371 | | 23.85 | 82.18 | -1.784798064 | -5.82235 | 5.80E-09 | TRUE | |
| cfa-miR-653 | | 47.69 | 11.74 | 2.022254374 | 4.8200425 | 1.44E-06 | TRUE | |
| cfa-miR-219-3p | | 0 | 35.22 | -6.138323004 | -6.163669 | 7.11E-10 | TRUE | |
| cfa-miR-1837 | | 0 | 11.74 | -4.553360503 | -3.474201 | 0.0005124 | TRUE | |
| cfa-miR-514 | | 0 | 11.74 | -4.553360503 | -3.474201 | 0.0005124 | TRUE | |
|  | |  |  |  |  |  |  | |
| **BAC2_vs_CFDAC2; 30 (22 up, 8 down) DE-miRNAs; P-value < 0.001, log2(Fold_change) >1** | | | | | | | | |
| **name** | | **BAC2** | **CFDAC2** | **log2(Fold_change)** | **z-score** | **p-value** | **Signature(p-value < 0.001)** | |
| cfa-miR-889 | | 677125.6 | 285996.41 | 1.243426422 | 109.57586 | 0 | TRUE | |
| cfa-miR-218 | | 588022.28 | 269087.97 | 1.127792925 | 349.43774 | 0 | TRUE | |
| cfa-miR-338 | | 168721.04 | 79629.01 | 1.083273867 | 181.04476 | 0 | TRUE | |
| cfa-miR-34a | | 60813.88 | 172140.37 | -1.501112932 | -235.2004 | 0 | TRUE | |
| cfa-miR-96 | | 72001.44 | 157603.3 | -1.130200078 | -180.6401 | 0 | TRUE | |
| cfa-miR-758 | | 78101.82 | 33774.31 | 1.209429872 | 134.50677 | 0 | TRUE | |
| cfa-miR-9 | | 68265.21 | 27267.15 | 1.323986609 | 134.94084 | 0 | TRUE | |
| cfa-miR-138a | | 35789.63 | 7958.84 | 2.16891155 | 138.19463 | 0 | TRUE | |
| cfa-miR-382 | | 21298.58 | 10638.86 | 1.001413679 | 60.301794 | 0 | TRUE | |
| cfa-miR-124 | | 24559.86 | 5837.16 | 2.072963818 | 111.2506 | 0 | TRUE | |
| cfa-miR-196b | | 1931.44 | 4344.88 | -1.169639471 | -30.82032 | 1.40E-208 | TRUE | |
| cfa-miR-138b | | 4242.83 | 984.7 | 2.107270711 | 46.724761 | 0 | TRUE | |
| cfa-miR-496 | | 2068.64 | 923.79 | 1.163045764 | 21.218739 | 6.41E-100 | TRUE | |
| cfa-miR-490 | | 2406.38 | 487.28 | 2.304041567 | 37.182476 | 1.31E-302 | TRUE | |
| cfa-miR-488 | | 1467.05 | 670 | 1.130685041 | 17.470024 | 2.42E-68 | TRUE | |
| cfa-miR-301b | | 1255.96 | 558.34 | 1.169574697 | 16.60739 | 6.16E-62 | TRUE | |
| cfa-miR-885 | | 1160.97 | 538.03 | 1.109572169 | 15.307131 | 6.85E-53 | TRUE | |
| cfa-miR-196a | | 84.43 | 1340.01 | -3.988344243 | -35.83934 | 2.70E-281 | TRUE | |
| cfa-miR-493 | | 833.79 | 294.4 | 1.501906398 | 16.399732 | 1.92E-60 | TRUE | |
| cfa-miR-34c | | 717.69 | 294.4 | 1.285583148 | 13.523175 | 1.14E-41 | TRUE | |
| cfa-miR-216b | | 517.16 | 203.03 | 1.348917776 | 11.912979 | 1.01E-32 | TRUE | |
| cfa-miR-491 | | 401.06 | 172.58 | 1.216552802 | 9.6820194 | 3.60E-22 | TRUE | |
| cfa-miR-1839 | | 63.33 | 131.97 | -1.059249023 | -4.957749 | 7.13E-07 | TRUE | |
| cfa-miR-514 | | 31.66 | 71.06 | -1.166376433 | -3.932676 | 8.40E-05 | TRUE | |
| cfa-miR-489 | | 73.88 | 20.3 | 1.863704139 | 5.6885666 | 1.28E-08 | TRUE | |
| cfa-miR-1836 | | 10.55 | 60.91 | -2.529436105 | -6.238659 | 4.41E-10 | TRUE | |
| cfa-miR-147 | | 42.22 | 10.15 | 2.056446851 | 4.5888585 | 4.46E-06 | TRUE | |
| cfa-miR-448 | | 21.11 | 0 | 5.399854674 | 4.7715997 | 1.83E-06 | TRUE | |
| cfa-miR-632 | | 21.11 | 0 | 5.399854674 | 4.7715997 | 1.83E-06 | TRUE | |
| cfa-miR-1840 | | 0 | 20.3 | -5.343407822 | -4.673447 | 2.96E-06 | TRUE | |
|  | |  |  |  |  |  |  | |
| **CFDAC1_vs_CFDAC2; 21 (15 up, 6 down) DE-miRNAs; P-value < 0.001, log2(Fold_change) >1** | | | | | | | | |
| **name** | | **CFDAC1** | **CFDAC2** | **log2(Fold_change)** | **z-score** | **p-value** | **Signature(p-value < 0.001)** | |
| cfa-miR-132 | | 64596.96 | 24262.28 | 1.412751141 | 138.00302 | 0 | TRUE | |
| cfa-miR-122 | | 16589.51 | 538.03 | 4.946440846 | 132.57468 | 0 | TRUE | |
| cfa-miR-592 | | 2031.13 | 4893.06 | -1.268454393 | -34.84685 | 4.75E-266 | TRUE | |
| cfa-miR-196a | | 399.18 | 1340.01 | -1.747132422 | -23.12907 | 2.36E-118 | TRUE | |
| cfa-miR-217 | | 962.73 | 355.31 | 1.438052954 | 17.0718 | 2.41E-65 | TRUE | |
| cfa-miR-216b | | 751.4 | 203.03 | 1.887888198 | 18.308465 | 7.08E-75 | TRUE | |
| cfa-miR-802 | | 727.92 | 81.21 | 3.16405061 | 24.198029 | 2.33E-129 | TRUE | |
| cfa-miR-491 | | 410.92 | 172.58 | 1.251592267 | 10.028244 | 1.15E-23 | TRUE | |
| cfa-miR-383 | | 93.93 | 223.33 | -1.249519148 | -7.357405 | 1.88E-13 | TRUE | |
| cfa-miR-184 | | 211.33 | 101.52 | 1.05773361 | 6.2873745 | 3.23E-10 | TRUE | |
| cfa-miR-208b | | 82.18 | 182.73 | -1.152854275 | -6.243066 | 4.29E-10 | TRUE | |
| cfa-miR-200b | | 129.15 | 30.45 | 2.08453351 | 8.0993268 | 5.53E-16 | TRUE | |
| cfa-miR-514 | | 11.74 | 71.06 | -2.59760528 | -6.836571 | 8.11E-12 | TRUE | |
| cfa-miR-1840 | | 58.7 | 20.3 | 1.531880776 | 4.4176729 | 9.98E-06 | TRUE | |
| cfa-miR-489 | | 46.96 | 20.3 | 1.209952681 | 3.3012276 | 0.0009626 | TRUE | |
| cfa-miR-1836 | | 0 | 60.91 | -6.928607199 | -7.975041 | 1.52E-15 | TRUE | |
| cfa-miR-147 | | 46.96 | 10.15 | 2.209952681 | 5.0661149 | 4.06E-07 | TRUE | |
| cfa-miR-219-3p | | 35.22 | 10.15 | 1.794915182 | 3.8300228 | 0.0001281 | TRUE | |
| cfa-miR-1837 | | 11.74 | 0 | 4.553360503 | 3.4754599 | 0.00051 | TRUE | |
| cfa-miR-448 | | 11.74 | 0 | 4.553360503 | 3.4754599 | 0.00051 | TRUE | |
| cfa-miR-615 | | 11.74 | 0 | 4.553360503 | 3.4754599 | 0.00051 | TRUE | |
|  | |  |  |  |  |  |  | |
|  | | | | | | |  |  |
