# Supplementary material for: Comparison of miRNA expression profiles in pituitary–adrenal axis between Beagle and Chinese Field dogs after chronic stress exposure
Source: PeerJ. 2016 Feb 18;4:e1682. doi: 10.7717/peerj.1682 (PMC4768678; doi:10.7717/peerj.1682)
Supplement: Table S8 [file peerj-04-1682-s011.docx]

**Table S8.** Differentially expressed miRNAs of BP1_vs_BP2, BP1_vs_CFDP1, BP2_vs_CFDP2, CFDP1_vs_CFDP2.

| **BP1_vs_BP2; 25 (19 up, 6 down) DE-miRNAs; P-value < 0.001, log2(Fold_change) >1** | | | | | | |
| --- | --- | --- | --- | --- | --- | --- |
| **name** | **BP1** | **BP2** | **log2(Fold_change)** | **z-score** | **p-value** | **Signature(p-value < 0.001)** |
| cfa-miR-146b | 109353.37 | 51298.66 | 1.092004635 | 146.19605 | 0 | TRUE |
| cfa-miR-205 | 10730.32 | 22724.29 | -1.082542119 | -66.44834 | 0 | TRUE |
| cfa-miR-2483 | 7604.603752 | 3522.326544 | 1.110344413 | 11.012099 | 0 | TRUE |
| cfa-miR-449a | 1828.340902 | 4749.323177 | -1.377186838 | -9.418547 | 2.29E-21 | TRUE |
| cfa-miR-8908a-3p | 4530.402235 | 1269.306863 | 1.835598255 | 12.463934 | 0 | TRUE |
| cfa-miR-187 | 2396.35 | 1109.35 | 1.111124029 | 21.944129 | 9.85E-107 | TRUE |
| cfa-miR-105a | 2223.28 | 1031.35 | 1.108155643 | 21.091105 | 9.60E-99 | TRUE |
| cfa-miR-448 | 1903.77 | 693.34 | 1.457224293 | 24.170394 | 4.56E-129 | TRUE |
| cfa-miR-139 | 1650.82 | 615.34 | 1.423727139 | 22.117248 | 2.16E-108 | TRUE |
| cfa-miR-224 | 625.71 | 1378.02 | -1.139030761 | -17.04675 | 3.70E-65 | TRUE |
| cfa-miR-8908d | 1650.360814 | 285.5940441 | 2.530743674 | 9.1850753 | 0 | TRUE |
| cfa-miR-8908a-5p | 1375.300678 | 349.0593872 | 1.978202651 | 7.2215017 | 5.14E-13 | TRUE |
| cfa-miR-219-3p | 1118.3 | 424.67 | 1.396893163 | 17.943296 | 5.41E-72 | TRUE |
| cfa-miR-8908b | 1164.960575 | 264.4389297 | 2.139274645 | 6.9891946 | 2.76E-12 | TRUE |
| cfa-miR-802 | 812.1 | 346.67 | 1.228094394 | 13.838844 | 1.49E-43 | TRUE |
| cfa-miR-653 | 612.4 | 199.34 | 1.619243049 | 14.813414 | 1.20E-49 | TRUE |
| cfa-miR-105b | 625.71 | 164.67 | 1.925916416 | 16.891555 | 5.19E-64 | TRUE |
| cfa-miR-147 | 119.82 | 277.34 | -1.210786969 | -8.02784 | 9.92E-16 | TRUE |
| cfa-miR-676 | 53.25 | 121.33 | -1.188082884 | -5.230944 | 1.69E-07 | TRUE |
| cfa-miR-489 | 119.82 | 34.67 | 1.789108997 | 7.0307614 | 2.05E-12 | TRUE |
| cfa-miR-329a | 106.5 | 26 | 2.034269902 | 7.2244086 | 5.03E-13 | TRUE |
| cfa-miR-424 | 79.88 | 34.67 | 1.204146496 | 4.2731379 | 1.93E-05 | TRUE |
| cfa-miR-371 | 26.63 | 0 | 5.734980523 | 5.3672439 | 7.99E-08 | TRUE |
| cfa-miR-1836 | 0 | 26 | -5.700439718 | -5.307404 | 1.11E-07 | TRUE |
| cfa-miR-1843 | 13.31 | 0 | 4.734438666 | 3.7252233 | 0.0001951 | TRUE |
|  |  |  |  |  |  |  |
| **BP1_vs_CFDP1; 58 (13 up, 45 down) DE-miRNAs; P-value < 0.001, log2(Fold_change) >1** | | | | | | |
| **name** | **BP1** | **CFDP1** | **log2(Fold_change)** | **z-score** | **p-value** | **Signature(p-value < 0.001)** |
| cfa-miR-374a | 7711742.07 | 2786702.77 | 1.468497667 | 1569.6809 | 0 | TRUE |
| cfa-miR-30a | 6285675.13 | 2553207.51 | 1.299756913 | 1289.0059 | 0 | TRUE |
| cfa-miR-9 | 788518.44 | 2270769.54 | -1.525964896 | -869.0837 | 0 | TRUE |
| cfa-miR-124 | 51801.06 | 1806444.56 | -5.124027549 | -1390.527 | 0 | TRUE |
| cfa-miR-144 | 561344.62 | 162115.95 | 1.791860701 | 482.51739 | 0 | TRUE |
| cfa-miR-128 | 105386.08 | 439480.59 | -2.060115129 | -468.9343 | 0 | TRUE |
| cfa-miR-142 | 67510.46 | 163126.55 | -1.272808656 | -202.3109 | 0 | TRUE |
| cfa-miR-222 | 17160.52 | 153481.79 | -3.16090232 | -351.1314 | 0 | TRUE |
| cfa-miR-221 | 10703.69 | 90709.51 | -3.083145573 | -266.8583 | 0 | TRUE |
| cfa-miR-190a | 55994.67 | 27136.89 | 1.045034116 | 101.04172 | 0 | TRUE |
| cfa-miR-95 | 23657.28 | 47877.85 | -1.017074159 | -91.54961 | 0 | TRUE |
| cfa-miR-138a | 10264.36 | 52944.4 | -2.366834421 | -177.2061 | 0 | TRUE |
| cfa-miR-149 | 17386.84 | 43659.12 | -1.3282873 | -108.1527 | 0 | TRUE |
| cfa-miR-34c | 53531.76 | 6619.75 | 3.015546447 | 202.7306 | 0 | TRUE |
| cfa-miR-155 | 12913.66 | 26499.33 | -1.037057935 | -69.20317 | 0 | TRUE |
| cfa-miR-135b | 28942.56 | 10065.27 | 1.523806662 | 97.514074 | 0 | TRUE |
| cfa-miR-383 | 8134.27 | 24389.97 | -1.5842032 | -92.21367 | 0 | TRUE |
| cfa-miR-1343 | 8219.444055 | 22031.37478 | -1.422446804 | -21.1927 | 5.58E-100 | TRUE |
| cfa-miR-885 | 7042.6 | 22660.42 | -1.685994552 | -92.93729 | 0 | TRUE |
| cfa-miR-1249 | 8478.324183 | 19678.59023 | -1.214775833 | -17.96139 | 1.95E-72 | TRUE |
| cfa-miR-150 | 3980.6 | 17024.14 | -2.096524109 | -93.27838 | 0 | TRUE |
| cfa-miR-105a | 2223.28 | 16712.15 | -2.910135788 | -111.4005 | 0 | TRUE |
| cfa-miR-326 | 5618.11 | 13307.32 | -1.244063275 | -56.74567 | 0 | TRUE |
| cfa-let-7d | 5549.742738 | 11281.72663 | -1.023495083 | -11.9869 | 2.08E-33 | TRUE |
| cfa-miR-330 | 3953.97 | 9454.84 | -1.257751119 | -48.24248 | 0 | TRUE |
| cfa-miR-455 | 8640.17 | 3337 | 1.372508012 | 49.258971 | 0 | TRUE |
| cfa-miR-219-3p | 1118.3 | 10445.09 | -3.223445755 | -92.39264 | 0 | TRUE |
| cfa-miR-1296 | 2993.301477 | 8039.373365 | -1.425345464 | -12.81885 | 6.43E-38 | TRUE |
| cfa-miR-328 | 3567.9 | 7338.69 | -1.040447376 | -36.5142 | 6.60E-292 | TRUE |
| cfa-miR-138b | 1570.94 | 8715.55 | -2.47196363 | -73.73035 | 0 | TRUE |
| cfa-miR-202 | 1397.87 | 7365.82 | -2.397615944 | -66.59583 | 0 | TRUE |
| cfa-miR-133a | 2050.21 | 5066.55 | -1.305232007 | -36.34647 | 2.99E-289 | TRUE |
| cfa-miR-133c | 2050.21 | 5066.55 | -1.305232007 | -36.34647 | 2.99E-289 | TRUE |
| cfa-miR-769 | 2087.22103 | 4472.784767 | -1.099589951 | -7.964274 | 8.31E-16 | TRUE |
| cfa-miR-139 | 1650.82 | 4883.42 | -1.564709041 | -40.89129 | 0 | TRUE |
| cfa-miR-224 | 625.71 | 2231.45 | -1.834415414 | -30.92333 | 5.80E-210 | TRUE |
| cfa-miR-345 | 878.66 | 1824.5 | -1.054124227 | -18.40164 | 1.27E-75 | TRUE |
| cfa-miR-8908d | 1650.360814 | 798.117728 | 1.048107996 | 5.2221006 | 1.77E-07 | TRUE |
| cfa-miR-1 | 439.33 | 1505.72 | -1.777076591 | -24.85396 | 2.34E-136 | TRUE |
| cfa-miR-105b | 625.71 | 1268.33 | -1.019364095 | -14.92684 | 2.20E-50 | TRUE |
| cfa-miR-8908a-5p | 1375.300678 | 515.4510327 | 1.415839781 | 6.1147371 | 9.67E-10 | TRUE |
| cfa-miR-8908b | 1164.960575 | 565.3333907 | 1.043107316 | 4.3694943 | 1.25E-05 | TRUE |
| cfa-miR-346 | 199.7 | 1329.38 | -2.734847316 | -30.43755 | 1.75E-203 | TRUE |
| cfa-miR-34b | 1331.3 | 115.3 | 3.529371292 | 34.230373 | 8.55E-257 | TRUE |
| cfa-miR-1307 | 306.2 | 874.95 | -1.514726292 | -16.90164 | 4.38E-64 | TRUE |
| cfa-miR-876 | 359.45 | 718.95 | -1.000100337 | -11.063 | 1.90E-28 | TRUE |
| cfa-miR-8903 | 242.7001197 | 814.7451807 | -1.747174094 | -4.639858 | 1.74E-06 | TRUE |
| cfa-miR-802 | 812.1 | 115.3 | 2.816264875 | 24.137368 | 1.01E-128 | TRUE |
| cfa-miR-208b | 559.15 | 223.82 | 1.320896401 | 12.16768 | 4.62E-34 | TRUE |
| cfa-miR-206 | 13.31 | 746.08 | -5.808747858 | -28.42554 | 9.78E-178 | TRUE |
| cfa-miR-491 | 119.82 | 257.74 | -1.105047715 | -7.186412 | 6.65E-13 | TRUE |
| cfa-miR-133b | 13.31 | 271.3 | -4.349306571 | -16.52865 | 2.28E-61 | TRUE |
| cfa-miR-1835 | 26.63 | 196.69 | -2.884799277 | -12.03275 | 2.39E-33 | TRUE |
| cfa-miR-1839 | 53.25 | 128.87 | -1.275063023 | -5.692465 | 1.25E-08 | TRUE |
| cfa-miR-764 | 0 | 115.3 | -7.849248703 | -10.60214 | 2.91E-26 | TRUE |
| cfa-miR-122 | 13.31 | 88.17 | -2.727777288 | -7.828044 | 4.96E-15 | TRUE |
| cfa-miR-371 | 26.63 | 61.04 | -1.196702534 | -3.727397 | 0.0001935 | TRUE |
| cfa-miR-632 | 0 | 13.57 | -4.762348816 | -3.767995 | 0.0001646 | TRUE |
|  |  |  |  |  |  |  |
| **BP2_vs_CFDP2; 32 (7 up, 25 down) DE-miRNAs; P-value < 0.001, log2(Fold_change) >1** | | | | | | |
| **name** | **BP2** | **CFDP2** | **log2(Fold_change)** | **z-score** | **p-value** | **Signature(p-value < 0.001)** |
| cfa-miR-146a | 181491 | 413207.36 | -1.186967944 | -304.6366 | 0 | TRUE |
| cfa-miR-1271 | 71492.25 | 149139.55 | -1.060804131 | -167.0953 | 0 | TRUE |
| cfa-miR-146b | 51298.66 | 163222.07 | -1.669843098 | -247.6139 | 0 | TRUE |
| cfa-miR-758 | 141892.49 | 68999.1 | 1.040148784 | 160.43721 | 0 | TRUE |
| cfa-miR-212 | 31720.41 | 67044.17 | -1.079700466 | -113.6382 | 0 | TRUE |
| cfa-miR-124 | 26407.67 | 67009.05 | -1.343398939 | -135.0442 | 0 | TRUE |
| cfa-miR-34c | 77871 | 12721.68 | 2.61379695 | 227.31677 | 0 | TRUE |
| cfa-miR-205 | 22724.29 | 55847.22 | -1.297250248 | -120.0029 | 0 | TRUE |
| cfa-miR-95 | 13823.51 | 31697.38 | -1.197239612 | -84.89128 | 0 | TRUE |
| cfa-miR-10a | 9186.79 | 31665.19 | -1.78526498 | -114.2687 | 0 | TRUE |
| cfa-miR-155 | 7254.09 | 14735.14 | -1.022394218 | -50.94481 | 0 | TRUE |
| cfa-miR-504 | 6144.75 | 13640.62 | -1.150482999 | -53.94721 | 0 | TRUE |
| cfa-miR-216b | 2080.03 | 5815.04 | -1.483184779 | -42.86602 | 0 | TRUE |
| cfa-miR-449a | 4749.323177 | 1402.595742 | 1.759622677 | 18.413081 | 0 | TRUE |
| cfa-miR-105a | 1031.35 | 3166.52 | -1.618364181 | -33.71615 | 3.35E-249 | TRUE |
| cfa-miR-8908a-3p | 1269.306863 | 2759.204739 | -1.12021162 | -8.074244 | 3.39E-16 | TRUE |
| cfa-miR-216a | 762.68 | 2718.76 | -1.833799029 | -34.10357 | 6.53E-255 | TRUE |
| cfa-miR-219-3p | 424.67 | 2774.36 | -2.707740902 | -43.72137 | 0 | TRUE |
| cfa-miR-215 | 754.01 | 1940.3 | -1.36362417 | -23.24134 | 1.74E-119 | TRUE |
| cfa-miR-34b | 1906.69 | 269.24 | 2.824105635 | 37.053119 | 1.60E-300 | TRUE |
| cfa-miR-1307 | 355.34 | 1375.48 | -1.952663158 | -25.30308 | 2.95E-141 | TRUE |
| cfa-miR-802 | 346.67 | 1085.75 | -1.647057053 | -19.99328 | 6.30E-89 | TRUE |
| cfa-miR-8908d | 285.5940441 | 942.7282858 | -1.722876124 | -6.246769 | 2.10E-10 | TRUE |
| cfa-miR-105b | 164.67 | 749.19 | -2.185753898 | -20.07605 | 1.20E-89 | TRUE |
| cfa-miR-8908b | 264.4389297 | 620.8210663 | -1.231242931 | -4.093337 | 2.13E-05 | TRUE |
| cfa-miR-507b | 497.1451878 | 195.443669 | 1.34691439 | 4.7868129 | 1.69E-06 | TRUE |
| cfa-miR-514 | 164.67 | 70.24 | 1.229212995 | 6.2488602 | 4.13E-10 | TRUE |
| cfa-miR-489 | 34.67 | 105.36 | -1.603567509 | -6.109514 | 9.99E-10 | TRUE |
| cfa-miR-424 | 34.67 | 70.24 | -1.018605008 | -3.506505 | 0.000454 | TRUE |
| cfa-miR-1835 | 17.33 | 79.02 | -2.188946192 | -6.525953 | 6.76E-11 | TRUE |
| cfa-miR-1836 | 26 | 64.38 | -1.308100954 | -4.100373 | 4.12E-05 | TRUE |
| cfa-miR-206 | 17.33 | 2.93 | 2.564299085 | 3.355259 | 0.0007929 | TRUE |
|  |  |  |  |  |  |  |
| **CFDP1_vs_CFDP2; 43 (32 up, 11 down) DE-miRNAs; P-value < 0.001, log2(Fold_change) >1** | | | | | | |
| **name** | **CFDP1** | **CFDP2** | **log2(Fold_change)** | **z-score** | **p-value** | **Signature(p-value < 0.001)** |
| cfa-miR-30a | 2553207.51 | 5289957.28 | -1.050945275 | -999.1681 | 0 | TRUE |
| cfa-miR-9 | 2270769.54 | 548039.03 | 2.050830747 | 1064.4863 | 0 | TRUE |
| cfa-miR-124 | 1806444.56 | 67009.05 | 4.752653215 | 1375.0415 | 0 | TRUE |
| cfa-miR-135a-5p | 561579.63 | 1131398.51 | -1.010544665 | -443.7258 | 0 | TRUE |
| cfa-miR-128 | 439480.59 | 86511.53 | 2.34483512 | 507.52403 | 0 | TRUE |
| cfa-miR-144 | 162115.95 | 341015.4 | -1.072810852 | -255.413 | 0 | TRUE |
| cfa-miR-222 | 153481.79 | 29888.78 | 2.36039158 | 300.99452 | 0 | TRUE |
| cfa-miR-221 | 90709.51 | 19203.97 | 2.239849224 | 224.1915 | 0 | TRUE |
| cfa-miR-205 | 12764.71 | 55847.22 | -2.129324705 | -170.6371 | 0 | TRUE |
| cfa-miR-138a | 52944.4 | 7942.64 | 2.736787577 | 192.01747 | 0 | TRUE |
| cfa-miR-1343 | 22031.37478 | 10113.25181 | 1.123312568 | 29.034716 | 0 | TRUE |
| cfa-miR-885 | 22660.42 | 9446.88 | 1.262264764 | 74.769099 | 0 | TRUE |
| cfa-miR-135b | 10065.27 | 21000.87 | -1.061063222 | -62.77784 | 0 | TRUE |
| cfa-miR-150 | 17024.14 | 4521.51 | 1.912705361 | 87.759732 | 0 | TRUE |
| cfa-miR-105a | 16712.15 | 3166.52 | 2.39992725 | 100.27969 | 0 | TRUE |
| cfa-miR-326 | 13307.32 | 4126.43 | 1.68925398 | 71.225413 | 0 | TRUE |
| cfa-miR-330 | 9454.84 | 3792.8 | 1.317789746 | 49.938807 | 0 | TRUE |
| cfa-miR-219-3p | 10445.09 | 2774.36 | 1.912598015 | 68.738214 | 0 | TRUE |
| cfa-miR-138b | 8715.55 | 1413.52 | 2.624299413 | 76.170523 | 0 | TRUE |
| cfa-miR-202 | 7365.82 | 1597.89 | 2.204678047 | 63.259415 | 0 | TRUE |
| cfa-miR-769 | 4472.784767 | 1854.798741 | 1.269910684 | 14.508489 | 0 | TRUE |
| cfa-miR-139 | 4883.42 | 1123.79 | 2.119519395 | 50.253606 | 0 | TRUE |
| cfa-miR-551b | 3682.91 | 1814.46 | 1.021305889 | 25.42228 | 1.43E-142 | TRUE |
| cfa-miR-490 | 2916.49 | 1436.93 | 1.021243345 | 22.621771 | 2.65E-113 | TRUE |
| cfa-miR-448 | 2645.19 | 918.93 | 1.52534448 | 29.497648 | 3.09E-191 | TRUE |
| cfa-miR-592 | 2238.23 | 992.1 | 1.173800844 | 22.186857 | 4.60E-109 | TRUE |
| cfa-miR-215 | 596.86 | 1940.3 | -1.700815256 | -27.36517 | 7.13E-165 | TRUE |
| cfa-miR-1 | 1505.72 | 687.74 | 1.130518352 | 17.657904 | 8.85E-70 | TRUE |
| cfa-miR-346 | 1329.38 | 234.12 | 2.505433465 | 28.991216 | 8.49E-185 | TRUE |
| cfa-miR-802 | 115.3 | 1085.75 | -3.235227535 | -29.83733 | 1.28E-195 | TRUE |
| cfa-miR-8903 | 814.7451807 | 264.4237875 | 1.623496946 | 7.5445826 | 4.53E-14 | TRUE |
| cfa-miR-206 | 746.08 | 2.93 | 7.99228586 | 26.781337 | 5.33E-158 | TRUE |
| cfa-miR-34b | 115.3 | 269.24 | -1.223500249 | -7.967376 | 1.62E-15 | TRUE |
| cfa-miR-133b | 271.3 | 23.41 | 3.534692208 | 15.460857 | 6.37E-54 | TRUE |
| cfa-miR-1835 | 196.69 | 79.02 | 1.315633859 | 7.1935859 | 6.31E-13 | TRUE |
| cfa-miR-514 | 189.91 | 70.24 | 1.434951125 | 7.5523544 | 4.27E-14 | TRUE |
| cfa-miR-147 | 81.39 | 172.67 | -1.085093995 | -5.79676 | 6.76E-09 | TRUE |
| cfa-miR-329a | 101.74 | 46.82 | 1.11969016 | 4.5545198 | 5.25E-06 | TRUE |
| cfa-miR-764 | 115.3 | 11.71 | 3.299579532 | 9.7997314 | 1.13E-22 | TRUE |
| cfa-miR-8875 | 74.823537 | 3.8322288 | 4.287236561 | 3.9772385 | 6.97E-05 | TRUE |
| cfa-miR-371 | 61.04 | 5.85 | 3.383246432 | 7.2059124 | 5.77E-13 | TRUE |
| cfa-miR-1836 | 0 | 64.38 | -7.008540672 | -8.18351 | 2.76E-16 | TRUE |
| cfa-miR-1840 | 6.78 | 32.19 | -2.247255398 | -4.235678 | 2.28E-05 | TRUE |
